# Genetic association analysis of 77,539 genomes reveals rare disease etiologies

**DOI:** 10.1038/s41591-023-02211-z

**Published:** 2023-03-16

**Authors:** Daniel Greene, Genomics England Research Consortium, Daniela Pirri, Karen Frudd, Ege Sackey, Mohammed Al-Owain, Arnaud P. J. Giese, Khushnooda Ramzan, Sehar Riaz, Itaru Yamanaka, Nele Boeckx, Chantal Thys, Bruce D. Gelb, Paul Brennan, Verity Hartill, Julie Harvengt, Tomoki Kosho, Sahar Mansour, Mitsuo Masuno, Takako Ohata, Helen Stewart, Khalid Taibah, Claire L. S. Turner, Faiqa Imtiaz, Saima Riazuddin, Takayuki Morisaki, Pia Ostergaard, Bart L. Loeys, Hiroko Morisaki, Zubair M. Ahmed, Graeme M. Birdsey, Kathleen Freson, Andrew Mumford, Ernest Turro

**Affiliations:** 1 Department of Medicine, University of Cambridge, Cambridge, UK; 2 Mindich Child Health and Development Institute, Icahn School of Medicine at Mount Sinai, New York, NY USA; 3 National Heart and Lung Institute, Imperial College London, London, UK; 4 University College London Institute of Ophthalmology, University College London, London, UK; 5 Molecular and Clinical Sciences Institute, St. George’s University of London, London, UK; 6 Department of Medical Genomics, Centre for Genomic Medicine, King Faisal Specialist Hospital & Research Centre, Riyadh, Saudi Arabia; 7 Department of Otorhinolaryngology Head and Neck Surgery, School of Medicine, University of Maryland, Baltimore, MD USA; 8 Department of Clinical Genomics, Centre for Genomic Medicine, King Faisal Specialist Hospital & Research Centre, Riyadh, Saudi Arabia; 9 Department of Biochemistry and Molecular Biology, School of Medicine, University of Maryland, Baltimore, MD USA; 10 Department of Bioscience and Genetics, National Cerebral and Cardiovascular Center, Osaka, Japan; 11 Center for Medical Genetics, Antwerp University Hospital/University of Antwerp, Antwerp, Belgium; 12 Department of Cardiovascular Sciences, Center for Molecular and Vascular Biology, KU Leuven, Leuven, Belgium; 13 Department of Pediatrics, Icahn School of Medicine at Mount Sinai, New York, NY USA; 14 Department of Genetics and Genomic Sciences, Icahn School of Medicine at Mount Sinai, New York, NY USA; 15 Northern Genetics Service, Newcastle upon Tyne Hospitals National Health Service Trust International Centre for Life, Newcastle upon Tyne, UK; 16 Department of Clinical Genetics, Chapel Allerton Hospital, Leeds Teaching Hospitals National Health Service Trust, Leeds, UK; 17 Leeds Institute of Medical Research, University of Leeds, Leeds, UK; 18 Centre for Medical Genetics, Centre Hospitalier Universitaire de Liège, Liège, Belgium; 19 Department of Medical Genetics, Shinshu University School of Medicine, Nagano, Japan; 20 Center for Medical Genetics, Shinshu University Hospital, Nagano, Japan; 21 South West Thames Regional Genetics Service, St. George’s University Hospitals National Health Service Foundation Trust, London, UK; 22 Department of Medical Genetics, Kawasaki Medical School Hospital, Okayama, Japan; 23 Okinawa Chubu Hospital, Okinawa, Japan; 24 Oxford University Hospitals National Health Service Foundation Trust, Oxford, UK; 25 Ear Nose and Throat Medical Centre, Riyadh, Saudi Arabia; 26 Peninsula Clinical Genetics Service, Royal Devon & Exeter Hospital, Exeter, UK; 27 Division of Molecular Pathology and Department of Internal Medicine, Institute of Medical Science, The University of Tokyo, Tokyo, Japan; 28 Department of Human Genetics, Radboud University Medical Center, Nijmegen, the Netherlands; 29 Department of Medical Genetics, Sakakibara Heart Institute, Tokyo, Japan; 30 School of Cellular and Molecular Medicine, University of Bristol, Bristol, UK; 31 South West National Health Service Genomic Medicine Service Alliance, Bristol, UK; 32 Department of Haematology, University of Cambridge, Cambridge Biomedical Campus, Cambridge, UK; 33 Charles Bronfman Institute for Personalized Medicine, Icahn School of Medicine at Mount Sinai, New York, NY USA

**Keywords:** Genetics, Genetics research, Computational platforms and environments, Next-generation sequencing, Disease genetics

## Abstract

The genetic etiologies of more than half of rare diseases remain unknown. Standardized genome sequencing and phenotyping of large patient cohorts provide an opportunity for discovering the unknown etiologies, but this depends on efficient and powerful analytical methods. We built a compact database, the ‘Rareservoir’, containing the rare variant genotypes and phenotypes of 77,539 participants sequenced by the 100,000 Genomes Project. We then used the Bayesian genetic association method BeviMed to infer associations between genes and each of 269 rare disease classes assigned by clinicians to the participants. We identified 241 known and 19 previously unidentified associations. We validated associations with *ERG*, *PMEPA1* and *GPR156* by searching for pedigrees in other cohorts and using bioinformatic and experimental approaches. We provide evidence that (1) loss-of-function variants in the Erythroblast Transformation Specific (ETS)-family transcription factor encoding gene *ERG* lead to primary lymphoedema, (2) truncating variants in the last exon of transforming growth factor-β regulator *PMEPA1* result in Loeys–Dietz syndrome and (3) loss-of-function variants in *GPR156* give rise to recessive congenital hearing impairment. The Rareservoir provides a lightweight, flexible and portable system for synthesizing the genetic and phenotypic data required to study rare disease cohorts with tens of thousands of participants.

## Abstract (web summary)

A flexible and compact database containing rare variant genotypes and phenotypes of 77,539 participants sequenced by the 100,000 Genomes Project enables the identification of new disease-causing genes.

## Main

Collectively, rare diseases affect 1 in 20 people^1^, but fewer than half of the approximately 10,000 cataloged rare diseases have a resolved genetic etiology^2^. Standardized genome sequencing (GS) of large, phenotypically diverse collections of patients with rare diseases enables etiological discovery across a wide range of pathologies^3–5^ while boosting genetic diagnostic rates for patients. The 100,000 Genomes Project (100KGP), the largest GS study of patients with rare diseases to date, sequenced 34,523 UK National Health Service patients with rare diseases and 43,016 of their unaffected relatives. The linked genetic and phenotypic data of 100KGP participants were then made available to researchers through a web portal called the Genomics England Research Environment. The scale and complexity of such large GS datasets and the hierarchical nature of patient phenotype coding^6^ induce numerous bioinformatic and statistical challenges. Most importantly, the full genotype data from GS studies of tens of thousands of individuals are typically stored in unmodifiable files many terabytes in size, leading to high storage and processing costs. Recently developed frameworks, such as Hail^7^ and OpenCGA^8^, afford greater flexibility. However, they are designed to capture genotypes for variants across the full minor allele frequency (MAF) spectrum, from rare (MAF < 0.1%) to common (MAF > 5%) variants. To accommodate large numbers of genotypes, they depend on distributed storage systems and require numerous software packages, hindering deployment. We developed a database schema, the ‘Rareservoir’, for working with rare variant genotypes and patient phenotypes flexibly and efficiently. We deployed a Rareservoir only 5.5 GB in size of 100KGP data and applied the Bayesian statistical method BeviMed^9^ to identify genetic associations between coding genes and each of the 269 rare disease classes assigned to patients by clinicians. Of the previously unknown associations that we identified, we followed up the most plausible subset in confirmatory analytical and experimental work.

## Results

### The Rareservoir

**Extended Data Fig. 1:**
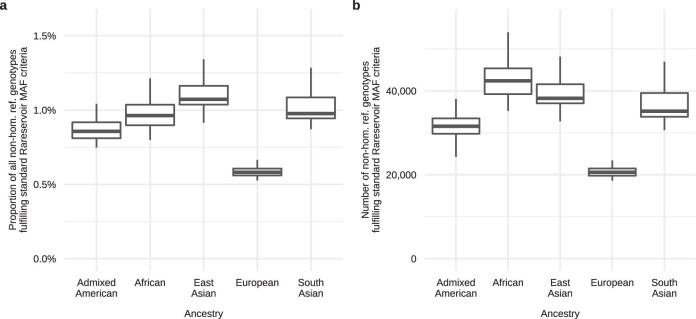
Reduction in the number of genotypes stored per sample. For 100 randomly chosen 100KGP participants belonging to each ancestry group (taken from amongst those with an inferred probability >0.9 of belonging): **a**, boxplots showing the distribution of the number of non-homozygous reference PASSing genotypes for variants on chromosomes 1–22 and X which meet the default Rareservoir MAF filtering criteria (that is a PMAF score >0 using gnomAD v3.0 and internal MAF < 0.002); **b**, boxplots showing the distribution of the proportion of all PASSing non-homozygous reference genotypes that meet the default Rareservoir MAF filtering criteria. In both plots, the lower, centre and upper lines respectively indicate the lower quartile, median and upper quartile. Whiskers are drawn up to the most extreme points that are less than 1.5× the interquartile range away from the nearest quartile.

**Extended Data Fig. 2:**
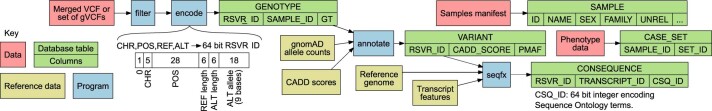
General schematic of the database build procedure and contents. Variants are extracted from VCF files, filtered on internal cohort allele frequency, encoded as 64-bit RSVR IDs and loaded into a table containing the corresponding genotypes. The variants are annotated with scores reflecting their predicted deleteriousness (in this case, CADD scores) and probabilistic minor allele frequency scores (PMAF) from gnomAD. The consequences of each variant with respect to a reference set of transcripts are generated and loaded into a table. Sample information including pedigree membership and membership of a maximal set of unrelated participants is loaded into a table. The case groupings for case/control association analyses are stored in a table.

**Extended Data Fig. 3:**
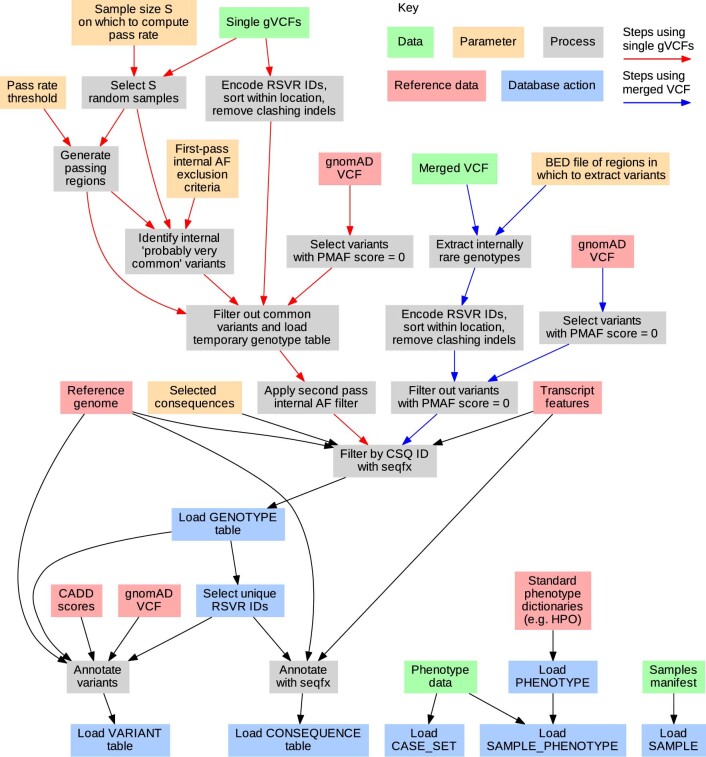
Detailed schematic of the database build procedure. Variants may be imported to a Rareservoir from either single gVCF files or a merged VCF file, following the procedures indicated by red and blue arrows respectively.

Relational databases (RDBs) provide a unified, centralized structure for storing, querying and modifying data of multiple underlying types. In principle, an RDB could provide a convenient foundation for the analysis of genotypes, variants, genes, participants and statistical results, but they cannot accommodate tables of the scale required to store exome or genome-wide genotypes in a moderately sized cohort. An RDB can, however, accommodate a sparse representation of genotypes corresponding to rare variants only, which encompass almost all variants having a large effect on rare disease risk. We developed an RDB schema, the Rareservoir, and complementary build procedure for the analysis of rare diseases, which by default, stores genotypes corresponding to variants for which all population-specific MAFs are likely to be <0.1%. This reduces the number of stored genotypes in a large study by about 99% (Extended Data Fig. 1). The Rareservoir encodes variants as 64-bit integers (‘RSVR IDs’) (Extended Data Fig. 2), which can represent 99.3% of variants encountered in practice without loss of information. RSVR IDs occupy a single column and increase numerically with respect to genomic position, allowing fast location-based queries within a simple database structure. To support the build process of a Rareservoir, we developed a complementary software package called ‘rsvr’ (Extended Data Figs. 2 and 3). The package includes tools to annotate variants with MAF information from control databases (for example, gnomAD^10^), pathogenicity scores (for example, combined annotation-dependent depletion (CADD) scores^11^) and predicted Sequence Ontology (SO)^12^ consequences with respect to a set of transcripts. We use a 64-bit integer (‘CSQ ID’) to record the consequences for interacting variant/transcript pairs, where each bit encodes one of the possible consequences, ordered by severity. Encoding the consequences in this way is efficient and enables succinct queries that threshold or sort based on severity of impact. The Rareservoir also contains a table with genetically derived data for each sample (including ancestry, sex and membership of a maximal set of unrelated participants) and a table of ‘case sets’ storing the rare disease classes assigned to each participant.

### BeviMed infers 241 known and 19 unknown genetic associations

**Fig. 1:**
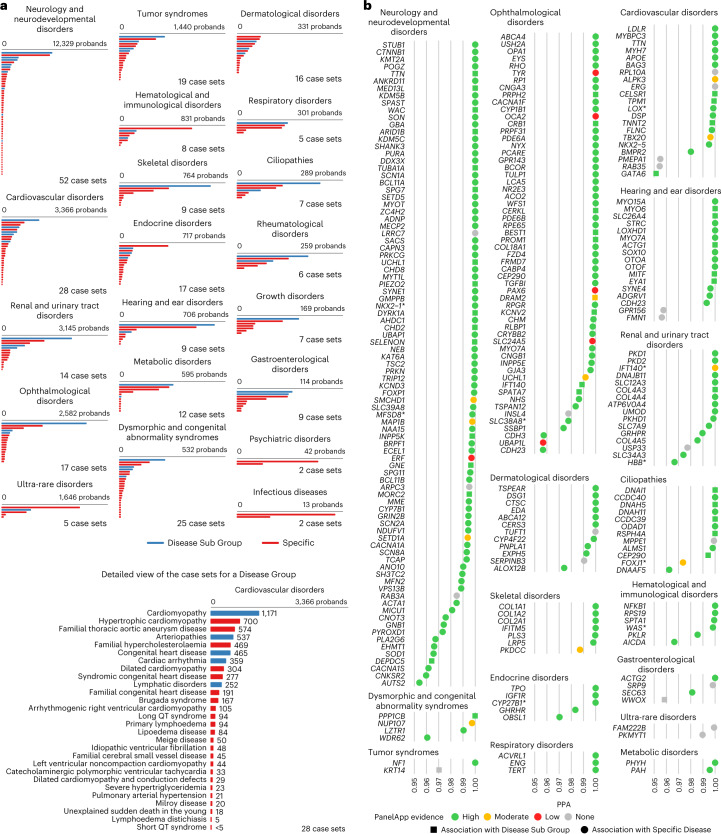
BeviMed analysis of the 100KGP. **a**, Bars showing the size of each case set used for the genetic association analyses grouped by Disease Group and coloured by type (Disease Sub Group or Specific Disease). Case sets smaller than five are shown as having size 4 to comply with the 100KGP policy on limiting participant identifiability. The names and sizes of the case sets for an exemplar Disease Sub Group, ‘Cardiovascular disorders’, are shown. **b**, BeviMed PPAs > 0.95 arranged by Disease Group. Only the strongest association for each gene within a Disease Group is shown. Associations are colored by their PanelApp evidence level (green, amber or red). Associations that were mapped to PanelApp by manual review, rather than using our automatic matching algorithm, are marked with an asterisk (Source Data Fig. 1). Previously unidentified associations are shown in grey. The shape of the points shows whether the association was with a Disease Sub Group (squares) or Specific Disease (circles). Source data

**Extended Data Fig. 4:**
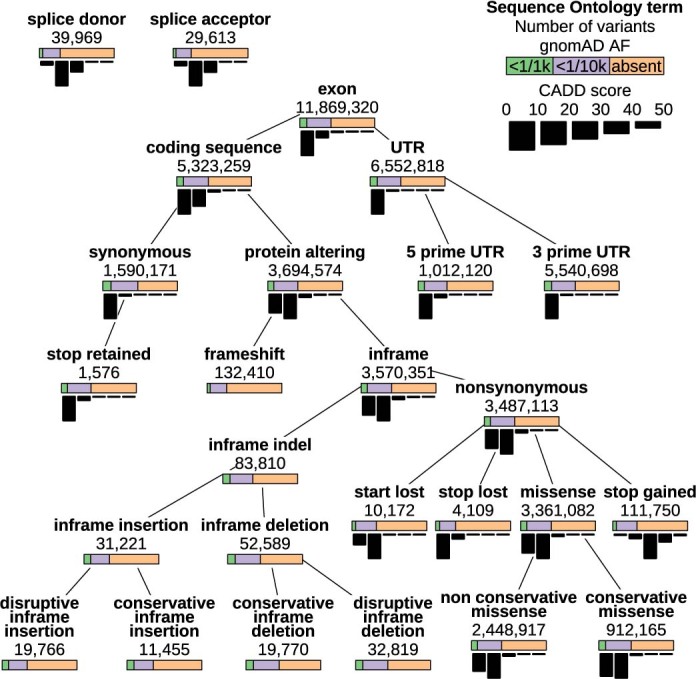
Schematic showing the variant data in the 100KGP Main Programme Rareservoir. The number of variant/transcript pairs, the distribution of CADD scores and a breakdown of gnomAD frequency classes is shown for each annotated SO term in the context of the structure of the ontology.

**Extended Data Fig. 5:**
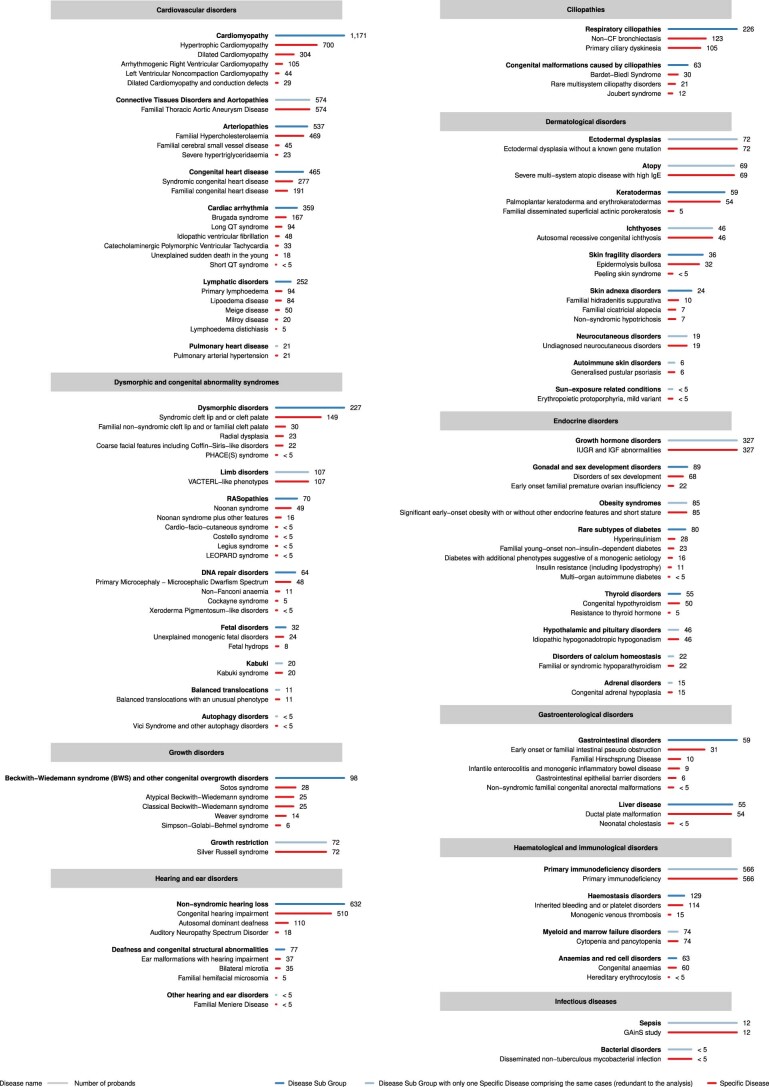
The 269 case sets, Disease Groups A–I. The names and sizes of the case sets used for the genetic association analyses, grouped by Disease Group and coloured by type (Disease Sub Group or Specific Disease). Disease Sub Groups with only one Specific Disease were excluded to avoid repeating identical analyses. Case sets smaller than 5 are labelled ‘<5’ and shown as having size 4 to comply with 100KGP policy on limiting participant identifiability. For legibility, only Disease Groups starting with the letters A–I are shown here.

**Extended Data Fig. 6:**
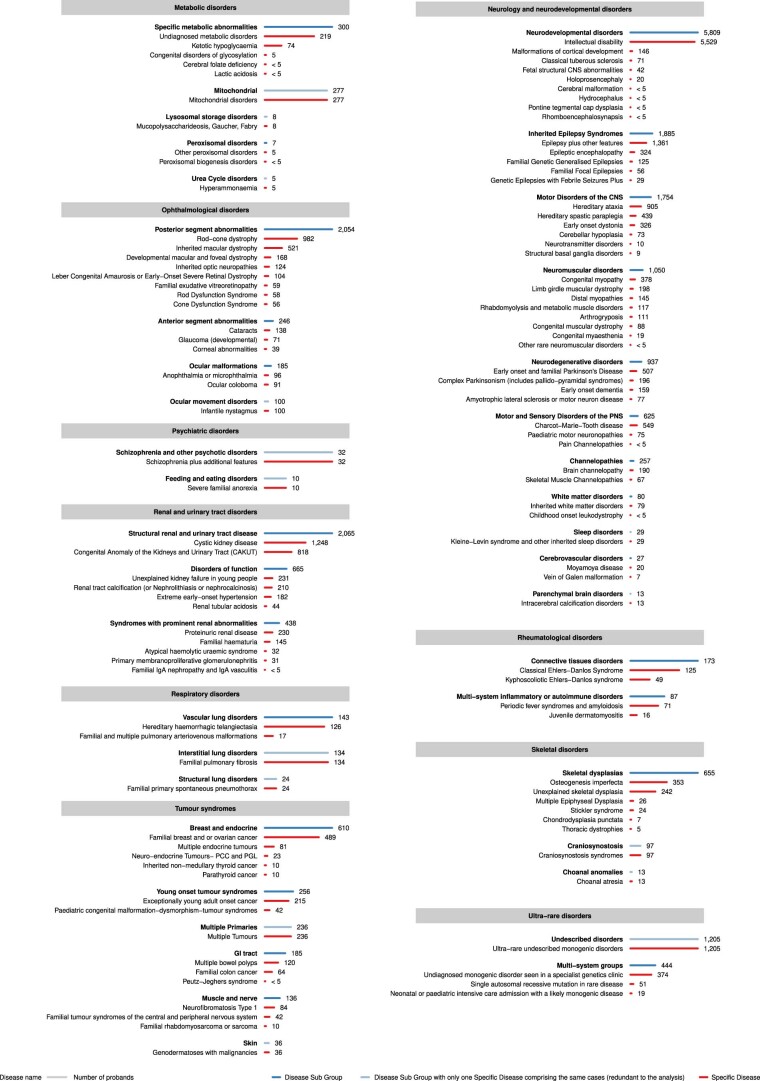
The 269 case sets, Disease Groups M–Z. An extension of Extended Data Fig. 5 showing the case sets in Disease Groups starting with the letters M–Z.

We built a Rareservoir, 5.5 GB in size, containing 11.9 million rare exonic and splicing single-nucleotide variants (SNVs) and short insertions or deletions (indels) affecting canonical transcripts of protein-coding genes in Ensembl v.104 (ref. ^13^) from a merged variant call format file (VCF) containing genotype calls for 77,539 participants, including 29,741 probands, in the Rare Diseases Main Programme of the 100KGP (Data Release v.13) (Extended Data Fig. 4). During enrollment to the 100KGP, expert clinicians used the clinical characteristics of each affected participant to assign them to one or more of 220 ‘Specific Diseases’. The Specific Diseases are hierarchically arranged into 88 ‘Disease Sub Groups’, each of which belongs to 1 of 20 ‘Disease Groups’. Whereas the eligibility criteria for many Specific Diseases aligned to the same or closely related rare diseases, for others such as ‘Intellectual disability’, the criteria were broader and encompassed diverse genetic etiologies. We generated 269 analytical case sets corresponding to all distinct Specific Diseases and Disease Sub Groups, ranging in size from 5,809 to one proband, and stored them in the Rareservoir (Fig. 1a and Extended Data Figs. 5 and 6). We included these two levels of the phenotyping hierarchy to account for heterogeneity in presentation or diagnosis among cases sharing the same genetic etiology, with the aim of boosting power to identify statistical genetic associations.

**Extended Data Fig. 7:**
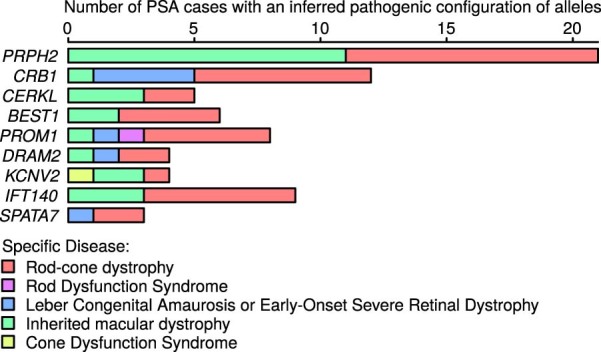
Breakdown of cases attributable to associations with ‘Posterior segment abnormalities’ by Specific Disease. For each gene associated with the Disease Sub Group ‘Posterior segment abnormalities’, a bar plot showing the number of cases having each of the different Specific Diseases who have an inferred pathogenic configuration of alleles in the gene. This example illustrates that sets of cases with the same etiological gene may be assigned different Specific Diseases. Consequently, pooling cases within Disease Sub Group can boost power.

Using the Bayesian statistical method BeviMed^9^, we obtained a posterior probability of association (PPA) between each of the 19,663 protein-coding genes and each of the 269 rare disease classes. BeviMed computes posterior probabilities over a baseline model of no association and competing association models, each of which assumes a particular mode of inheritance (MOI; dominant or recessive) and consequence class of etiological variant (in this study, high impact, moderate impact or 5′ untranslated region (UTR)). The PPA is obtained by summing the posterior probabilities over all association models. The association model with the greatest posterior probability (the modal model) determines the inferred MOI and class of etiological variant. Conditional on an association model, BeviMed models the pathogenicity of each included rare variant. In the model, participants with at least one pathogenic allele (under a dominant MOI) or at least as many pathogenic alleles as the ploidy (under a recessive MOI) have a pathogenic configuration of alleles, which determines their risk of case status. For each rare disease class, we selected a set of unrelated cases based on pedigree information provided by the 100KGP and compared them with participants not in the case set who belonged to different pedigrees and to a maximal set of unrelated participants, also provided by the 100KGP. To account for correlation between case sets, we only recorded the association for each gene having the highest PPA within a given Disease Group. Using a significance threshold of PPA > 0.95, we identified 260 significant associations, 241 of which were documented by the PanelApp gene panel database^14^, an expert-curated and annotated resource containing gene lists with high, medium or low levels of prior supporting evidence of causality for rare diseases (Fig. 1b). Of the 241 known associations that we identified, 43 (17.8%) were with Disease Sub Groups. For example, within each of the nine known genes associated with the Disease Sub Group ‘Posterior segment abnormalities’, the set of cases explained by variants with a conditional posterior probability of pathogenicity > 0.8 comprised participants encompassing multiple Specific Diseases (Extended Data Fig. 7). This demonstrates that participants with different Specific Diseases belonging to the same Disease Sub Group sometimes share defects in the same gene, which confirms that treating Disease Sub Groups, not just Specific Diseases, as case sets boosts statistical power.

Of the 241 associations identified as previously known according to PanelApp, 237 (98.3%) had an inferred MOI that was consistent with the MOIs listed for the relevant gene. Of these, the consistent MOI was found in the matched panel (223 associations), in the notes for the matched panel (5 associations) or in the MOIs listed for an alternative relevant panel (9 associations) in PanelApp (Source Data Fig. 1). This provided independent evidence that the genetic associations we labeled as known (without reference to MOI information) are genuinely supported by evidence in the literature, further demonstrating the accuracy of BeviMed’s inference. Of the four known associations with an inferred MOI that was incongruous with PanelApp, two had supporting evidence for the inferred MOI in the literature that was absent from PanelApp: *EDA* with dominant ‘Ectodermal dysplasia without a known gene mutation’^15^ and *AICDA* with dominant ‘Primary immunodeficiency’^16^. The two associations with an MOI that was unsupported in the literature were between *UCHL1* and dominant ‘Inherited optic neuropathies’ and between *SLC39A8* and dominant ‘Intellectual disability’.

Among 5,253 of the probands included in our analysis, the table of clinically reported variants available from the 100KGP Rare Diseases Main Programme at the time of this study comprised 4,907 distinct variants that had been classified as pathogenic or likely pathogenic in 1,863 genes. For 855 of these genes, etiological variants had been reported for only one family, suggesting that many genes that are etiological in the 100KGP are not identifiable by statistical association. Nevertheless, across the 260 associations identified by BeviMed, 2,536 distinct rare variants had a posterior probability of pathogenicity > 0.8 conditional on the modal model and were observed as part of a pathogenic configuration of alleles in a case (Source Data Fig. 1). Interestingly, among the subset of 2,485 variants contributing to the 241 known associations, only 1,604 featured in the table of clinically reported variants.

We found 19 previously unidentified genetic associations. To select a shortlist for further investigation, we assigned a plausibility score (range 0–3) based on three sources of additional evidence (Table 1). First, we considered evidence of purifying selection from gnomAD v.2.1.1. Any dominant associations with high-impact variants in a gene having a probability of loss-of-function intolerance (pLI) >0.9 or with moderate-impact variants in a gene having a *Z* score >2 were considered to be supported by population genetic metrics of purifying selection. To avoid disadvantaging recessive associations, which are unlikely to leave a detectable signature of purifying selection in gnomAD even if genuine, they were considered to be supported by default. Second, we considered cosegregation data: any association for which variants having a posterior probability of pathogenicity conditional on the modal model >0.8 tracked with case status in at least three additional family members and for which no affected relatives lacked the pertinent variants were considered to be supported by cosegregation. Third, we performed a comprehensive review of the literature for each gene and made a subjective assessment of whether an association was supported by biological function or previously known disease associations for related genes. In total, three genetic associations had a plausibility score of three and were, therefore, investigated further by gathering additional experimental evidence and looking for replication in other sequenced rare disease collections.

**Table 1:** Plausibility scoring of the 19 previously unknown genetic associations identified by BeviMed

Gene	Case set	Level	Cases	Controls	Variant class	MOI	pLI	*Z*	Cosegregation evidence	Biological function and existing disease associations	Score	Replication
*ERG*	Primary lymphoedema	SD	94	55,400	High	Dom	**0.96**	2.53	**Cosegregation in 2 affected and 1 unaffected relatives (mosaicism in 1 affected parent)**	**ETS-family transcription factor ERG is a critical regulator of endothelial lineage specification, vascular development, angiogenesis, and endothelial homeostasis**^20,40^	3	Internal (case enrolled for a different SD)
*GPR156*	Congenital hearing impairment	SD	510	54,739	High	**Rec**	0	1.04	**Cosegregation in 2 affected and 4 unaffected relatives**	**G protein-coupled receptor that regulates hair cell orientation in mechanosensory epithelia including in murine auditory epithelium**^33^	3	Riyadh
*PMEPA1*	FTAAD	SD	574	54,858	High	Dom	**0.94**	1.21	**Cosegregation in 3 affected relatives and distinctive phenotypic features**	**Negative regulator of TGFβ signaling**^28^**; aberrant TGFβ signaling is implicated in multiple Mendelian aortopathies**^29^	3	100KGP pilot, Antwerp, Tokyo
*FMN1*	Congenital hearing impairment	SD	510	54,738	High	**Rec**	0	-1.53	Cosegregation in 2 unaffected relatives.	**Formin family protein involved in linear actin and microtubule polymerization**^44^**; pathogenic variants in the formin DIAPH1 cause hearing loss via cytoskeletal disruption in auditory stereocilia**^45^	2	
*LRRC7*	Intellectual disability	SD	5,529	46,401	High	Dom	**1.00**	3.60		**Brain-specific scaffold protein in postsynaptic densities**^37^**; LRRC7-inactivated mice have a neurobehavioral phenotype**^38^	2	
*TUFT1*	Epidermolysis bullosa	SD	32	55,459	High	**Rec**	0	0.90	**Cosegregation in 1 affected and 4 unaffected relatives**	Acidic protein that mediates dental enamel mineralisation.	2	
*USP33*	Extreme early-onset hypertension	SD	182	55,305	High	Dom	0.86	**2.10**		**Deubiquitinating enzyme implicated in multiple cellular processes, including regulation of expression of the β2-adrenergic receptor**^39^**, a critical regulator of circulatory function and blood pressure**^41^	2	
*ARPC3*	Charcot-Marie-Tooth disease	SD	549	54,856	Moderate	Dom	0.22	0.39		**Component of the Arp2/3 complex that regulates polymerization of F-actin, abundant in axonal neurofilaments; multiple Mendelian axonal filamentopathies manifest as Charcot-Marie-Tooth disease**^42^**, and** ***Arpc3*-inactivation in mice causes axon dysfunction**^43^	1	
*KRT14*	Young-onset tumor syndromes	DSG	256	55,207	Moderate	**Rec**	0	0.84	Cosegregation in 2 unaffected relatives	Component of keratin intermediate filaments in epithelial cells; pathogenic variants cause Epidermolysis bullosa simplex (Dom/Rec), dermatopathia pigmentosa reticularis (Dom), and Naegeli-Franceschetti-Jadassohn syndrome (Dom)	1	
*MPPE1*	Primary ciliary dyskinesia	SD	105	55,360	High	**Rec**	0	0.35	Cosegregation in 2 unaffected relatives	Metallophosphoesterase required for transport of glycosylphosphatidylinositol-anchor proteins from the endoplasmic reticulum to the Golgi	1	
*PKMYT1*	Single autosomal recessive mutation in rare disease	SD	51	55,429	Moderate	**Rec**	0.22	0.07	Cosegregation in 2 unaffected relatives	Serine/threonine protein kinase that is a negative regulator of cell entry into mitosis	1	
*RAB35*	Familial hypercholesterolemia	SD	469	55,033	High	Dom	**0.98**	2.36	Cosegregation in 1 affected relative	Small GTP-binding proteins that are a regulator of endosomal transport and function	1	
*RAB3A*	Hereditary ataxia	SD	905	54,504	Moderate	Dom	0.95	**2.32**		Small GTP-binding proteins that regulate exocytosis and secretion; although abundant in brain synaptic vesicles, *Rab3A*-inactivated mice have no neuromuscular phenotype^46^	1	
*SERPINB3*	Autosomal recessive congenital ichthyosis	SD	46	55,437	Moderate	**Rec**	0	-1.66	Cosegregation in 2 unaffected relatives	Cysteine endopeptidase inhibitor implicated in autocrine/paracrine signaling and cell protein metabolism	1	
*WWOX*	Gastrointestinal disorders	DSG	59	55,413	Moderate	**Rec**	0	-4.44	Co-segregation in 1 unaffected relative	Short-chain dehydrogenase/reductase that acts as a tumor suppressor and apoptosis regulator; pathogenic variants cause developmental and epileptic encephalopathy 28 and spinocerebellar ataxia 12	1	
*FAM222B*	Ultra-rare undescribed monogenic disorders	SD	1,205	53,681	Moderate	Dom	0.29	0.42		Uncharacterized nucleosomal protein	0	
*INSL4*	Rod Dysfunction Syndrome	SD	58	55,425	Moderate	Dom	0	-1.43		Insulin-like growth factor implicated in trophoblast and bone development	0	
*RPL10A*	Milroy disease	SD	20	55,470	High	Dom	0.85	2.06		Component of the large ribosomal subunit that mediates protein translation	0	
*SRP9*	Ductal plate malformation	SD	54	55,445	High	Dom	0.42	1.13		Component of the signal recognition particle that targets secretory proteins to the endoplasmic reticulum	0	

Each row corresponds to a genetic association between a gene and a case set in the 100KGP Main Programme without prior supporting evidence in PanelApp. Each column gives additional information for each association. Cells contributing to the final score are shown in bold. Rows are sorted by score in descending order, and the genes corresponding to associations with a score of three are *ERG*, *GPR156* and *PMEPA1*. The level of the case set in the disease label hierarchy (Disease Sub Group (DSG), Specific Disease (SD)), the class of variants and the MOI corresponding to the model with the greatest posterior probability are shown (dominant (Dom), recessive (Rec)). A recessive association contributes one point to the score. A pLI > 0.9 contributes one point to the score providing the inferred class of etiological variants is high-impact variants. A *Z* score > 2 contributes one point to the score providing the inferred class of etiological variants is moderate-impact variants. Evidence of cosegregation in three or more relatives in the 100KGP data contributes one point to the score (including mosaicism supported by two or more reads containing the alternate allele). Prior evidence of a relevant biological function or disease association contributes one point to the score. The ‘Replication’ column specifies cohorts in which additional cases were confirmed.

### Variants in *ERG* are responsible for primary lymphoedema

**Fig. 2:**
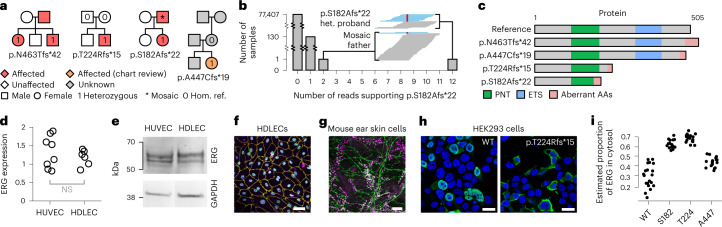
Loss-of-function variants in *ERG* are responsible for primary lymphoedema. **a**, Pedigrees for the four probands with loss-of-function variants in the canonical transcript of *ERG*, ENST00000288319.12. Hom. ref., homozygous reference. **b**, Truncated bar chart showing the distribution of the number of reads supporting the p.S182Afs*22 alternate allele in the 100KGP. The embedded windows show the read pileups at this position in the two affected members of the family with the variant encoding p.S182Afs*22 (het., heterozygous genotype call). The reads supporting the reference allele are in blue and those supporting the variant allele are in red. **c**, Schematic showing the effects of each variant at the cDNA and amino acid level and on the protein product with respect to the canonical transcript. PNT, pointed domain; ETS, Erythroblast Transformation Specific DNA binding domain; AA, amino acid. **d**, Reverse transcription PCR amplification of ERG mRNA in HDLECs relative to HUVECs. Data are normalized to GAPDH. Statistical significance was assessed using a two-sided Student’s *t* test. NS, not significant (*P* = 0.39). **e**, Immunoblot (representative of two replicates) of HUVEC and HDLEC protein lysates identified several bands corresponding to ERG isoforms expressed at similar intensities in both cell types. **f**, Immunofluorescence microscopy (representative of three replicates) of HDLECs shows ERG (green) nuclear colocalization with the lymphatic endothelial cell nuclear marker PROX1 (violet) and the nuclear marker DAPI (blue). HDLEC junctions are shown using an antibody to VE-cadherin (yellow). Scale bar, 50 µm. **g**, En face immunofluorescence confocal microscopy (representative of five replicates) of mouse ear skin. Vessels are stained with antibodies to the lymphatic marker PROX1 (violet) and ERG (green). Scale bar, 100 µm. **h**, Exemplar immunofluorescence microscopy image of HEK293 cells overexpressing wild-type *ERG* and the p.T224Rfs*15 variant *ERG*. Cells were stained for ERG (green) and nuclear marker DAPI (blue). Scale bars, 20 μm. The brightness is optimized for print. **i**, Dot plot of the estimated proportion of ERG not overlapping the nuclear marker DAPI in each of a set of immunofluorescence microscopy images of HEK293 cells overexpressing different *ERG* cDNAs (20 replicates for the wild type (WT), 17 replicates per tested mutant). The estimated proportions were significantly higher in each of the variants compared with WT: *P* = 1.52 × 10^−11^, 4.10 × 10^−13^ and 3.03 × 10^−5^ for each of p.S182Afs*22, p.T224Rfs*15 and p.A447Cfs*19, respectively (two-sided Student’s *t* tests). Source data

BeviMed identified a dominant genetic association between high-impact variants in *ERG* and the Specific Disease ‘Primary lymphoedema’, a group of genetic conditions caused by abnormal development of lymphatic vessels or failure of lymphatic function^17,18^. Three such variants were responsible for the high PPA, with locations ranging from codon 182 to 463 on the canonical Ensembl transcript ENST00000288319.12. One of the probands had two unaffected parents without the variant allele—one sequenced by the 100KGP and the other by Sanger sequencing—suggesting that the truncating heterozygous variant had appeared de novo. A participant in a fourth family who had been enrolled to the 100KGP for an unrelated condition also carried a predicted loss-of-function variant in *ERG*. Upon manual chart review, this participant had features associated with this unrelated condition but additional features consistent with primary lymphoedema, providing internal replication within the discovery cohort (Fig. 2a).

The affected father of the proband with the variant encoding p.S182Afs*22 was called homozygous for the reference allele, initially suggesting a lack of cosegregation of the variant with the disease in that pedigree. However, a review of the GS read alignments for the father revealed that 2 of the 48 reads overlapping that position supported the alternative allele. Specifically, these reads contained a deletion of a single G within the central poly-G tract of the motif ‘AGCTGGGGGTGAG’. To assess whether this could be the result of erroneous sequencing, we counted the number of such reads in the 77,539 genomes in the 100KGP and found that the proband and the father were the only two with more than one such read. This indicated that these reads in the father were unlikely to be erroneous but instead, that he was mosaic (Fig. 2b), consistent with the observation that his lymphoedema became clinically apparent over two decades later than his daughter, indicating milder disease. A further 130 samples collected through the 100KGP had a single read containing the deletion. This number was consistent with observations in the 80 other exonic loci that contain the same 13-base pair (bp) motif (mean 99.67 samples, range 4–149 samples), suggesting that, rather than being mosaic, the 130 samples contained individual sequencing errors. Furthermore, none of the participants who gave these samples had been assigned the Specific Disease ‘Primary lymphoedema’.

**Extended Data Fig. 8:**
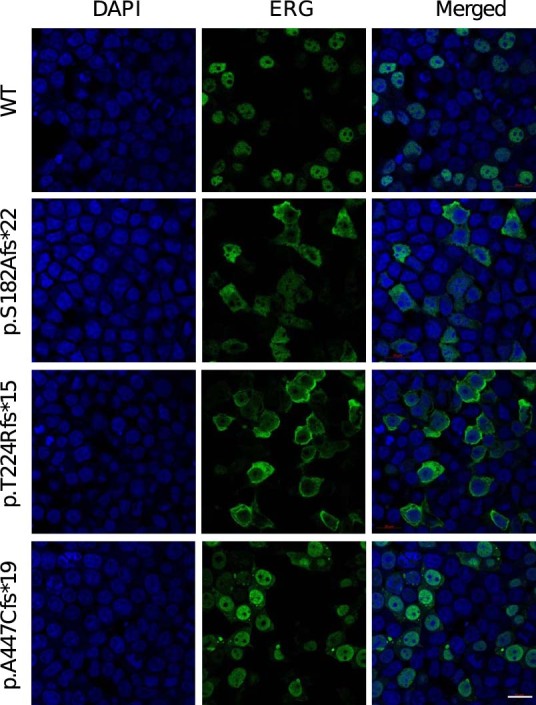
Microscopy images of HEK293 cells overexpressing ERG. Exemplar immunofluorescence microscopy images of HEK293 cells overexpressing wild type ERG (from 20 replicates) and each of the p.S182Afs*22, p.T224Rfs*15 and p.A447Cfs*19 variants of ERG (each from 17 replicates). Cells were stained for ERG (green) and nuclear marker DAPI (blue). Scale bar, 20μm.

*ERG* encodes a critical transcriptional regulator of blood vessel endothelial cell gene expression^19^ that is essential for normal vascular development^20^. However, little is known about the contribution of ERG to lymphatic development or how primary lymphoedema could arise from loss-of-function *ERG* variants that affect different parts of the ERG protein (Fig. 2c). Total cellular expression of ERG detected by real-time quantitative polymerase chain reaction (PCR) in purified RNA and by immunoblotting of protein extracts was the same in primary human dermal lymphatic endothelial cell (HDLECs) as human umbilical vein endothelial cell (HUVEC) (Fig. 2d,e, respectively). Moreover, immunofluorescence microscopy of cultured HDLECs showed that ERG expression colocalized with the lymphatic endothelial cell nuclear marker PROX1 (Fig. 2f), a finding confirmed in vivo by immunostaining whole mounts of ear skin from mice at 3 weeks after birth (Fig. 2g). The positions of the p.S182Afs*22 and p.T224Rfs*15 variants suggest nonsense-mediated decay and haploinsufficiency as a possible disease mechanism. The other two variants, however, are located in the final exon of ERG and may, therefore, evade nonsense-mediated decay. We studied both types of variant in more detail to explore potential disease mechanisms. In HEK293 cells, which do not express endogenous ERG, overexpression of wild-type ERG cDNA recapitulated the nuclear expression pattern observed in the HDLEC and mouse ear skin models. However, overexpression of ERG mutant cDNAs resulted in mislocalization of ERG outside of the nucleus, in the cytosol (Fig. 2h,i and Extended Data Fig. 8), preventing it from binding to DNA and exerting its function as a transcription factor^21^. Together, these data confirm high levels of ERG expression within the nuclei of the lymphatic endothelium consistent with a transcription regulatory function during lymphangiogenesis. They also suggest that in the primary lymphoedema cases, defective lymphangiogenesis may result from reduced ERG availability in the nucleus because of either haploinsufficiency resulting from nonsense-mediated decay or mislocalization.

### Variants in *PMEPA1* result in Loeys–Dietz syndrome

BeviMed identified a dominant genetic association between high-impact variants in *PMEPA1* and the Specific Disease ‘Familial thoracic aortic aneurysm disease’ (FTAAD). The variant with the highest conditional probability of pathogenicity was an insertion of one cytosine within a seven-cytosine stretch in the last exon of the canonical Ensembl transcript ENST00000341744.8. This variant, which is predicted to induce a p.S209Qfs*3 frameshift, was observed in three FTAAD pedigrees of European ancestry in the 100KGP discovery cohort. We replicated the association in three additional collections of cases. First, the same variant was identified independently in eight affected members of three pedigrees of Japanese ancestry in a separate Japanese patient group. Second, a single-cytosine deletion within the same polycytosine stretch as the previous variant, and encoding p.S209Afs*61, was found in an FTAAD case enrolled in a separate collection of 2,793 participants in the 100KGP Pilot Programme. Lastly, we identified a family in Belgium wherein the affected members carried a 5-bp deletion in the same stretch of polycytosines inducing a frameshift two residues upstream of the other two variants (p.P207Qfs*3).

**Fig. 3:**
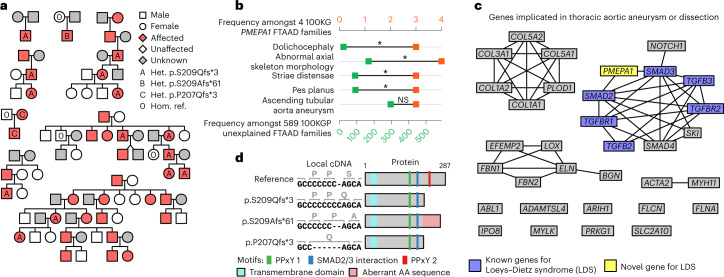
Truncating variants in *PMEPA1* result in Loeys–Dietz syndrome. **a**, Pedigrees for the three probands in the 100KGP (discovery cohort) heterozygous for the frameshift insertion predicting p.S209Qfs*3 and probands from replication cohorts, including one from the 100KGP Pilot Programme heterozygous for the frameshift deletion predicting p.S209Afs*61, three of Japanese ancestry heterozygous for p.S209Qfs*3 and one Belgian pedigree heterozygous for a frameshift deletion encoding p.P207Qfs*3. All variant consequences are shown with respect to the canonical transcript of *PMEPA1*, ENST00000341744.8. **b**, HPO terms present in at least three of the four *PMEPA1* FTAAD families, excluding redundant terms within each level of frequency, alongside their frequency in four *PMEPA1* FTAAD families and the other 589 unexplained FTAAD families. Terms are ordered by *P* values obtained by a Fisher exact test of association between the term’s presence in an FTAAD family and whether the family is one of the four *PMEPA1* families. Terms were declared significant (indicated by an asterisk) or not significant (NS) by comparing their Fisher test *P* values and rank with a null distribution of equivalent pairs obtained by permutation (10,000 replicates). For each rank, the *P* value of the term on the fifth percentile was used as an upper bound for declaring an association significant, provided all terms at higher ranks were also significant. The *P* values for each term were as follows: ‘Dolichocephaly’, *P* = 2.9 × 10^−4^; ‘Abnormal axial skeleton morphology’, *P* = 6.7 × 10^−3^; ‘Striae distensae’, *P* = 0.013; ‘Pes planus’, *P* = 0.014; ‘Ascending tubular aorta aneurysm’, *P* = 0.62. **c**, Graph showing *PMEPA1* and genes with high evidence (green) of association with FTAAD in PanelApp. Edges connect genes where the string-db v.11.5^27^ confidence score for physical interactions between corresponding proteins was >0.6. Genes known to be associated with Loeys–Dietz syndrome are highlighted in blue. *PMEPA1* is highlighted yellow. **d**, Schematic showing the effects of each variant at the cDNA and amino acid level and on the protein product.

All pedigrees exhibited dominant inheritance of aortic aneurysm disease with incomplete penetrance and skeletal features including pectus deformity, scoliosis and arachnodactyly with complete penetrance, which cosegregated with the respective variants in genotyped participants (Fig. 3a). To assess whether *PMEPA1* families affected by FTAAD form a phenotypically distinct subgroup, we analyzed the Human Phenotype Ontology (HPO) terms assigned to the 593 FTAAD families in both programs of the 100KGP. Using a permutation-based method^22,23^ based on the semantic similarity measure of Resnik et al.^24^, we found that the four 100KGP *PMEPA1* families were significantly more similar to each other than to other FTAAD families chosen at random (*P* = 5.7 × 10^−3^). To characterize the *PMEPA1* phenotype in greater detail, we compared the prevalence of each of the HPO terms in the minimal set of terms present in at least three of the four families with the prevalence in the other FTAAD families. We identified four HPO terms related to the musculoskeletal system that were significantly enriched (Fig. 3b), echoing the phenotypic characteristics of the syndromic aortopathy Loeys–Dietz syndrome^25,26^.

To understand the molecular mechanisms underlying this defect, we examined the protein–protein interactions^27^ for *PMEPA1* and the complete set of high-confidence genes in the ‘Thoracic aortic aneurysm or dissection’ PanelApp panel. *PMEPA1* encodes a negative regulator of transforming growth factor-β (TGFβ) signaling^28^, a pathway previously implicated in multiple aortopathies, including Loeys–Dietz syndrome^29^. The genes underlying known forms of Loeys–Dietz syndrome encode part of a tightly interacting subgroup of proteins in the TGFβ pathway, in which there is a direct interaction between the proteins encoded by *SMAD2*, *SMAD3* and *PMEPA1* (Fig. 3c). As the two candidate variants occur in the last exon of the transcript, they are likely to evade nonsense-mediated decay^30^. However, their truncating effects are predicted to remove a PPxY interaction motif while leaving the SMAD interaction motif intact (Fig. 3d), possibly affecting binding between PMEPA1 and SMAD2/3 and altering TGFβ signaling through a gain-of-function mechanism.

### Variants in *GPR156* lead to recessive congenital hearing loss

**Fig. 4:**
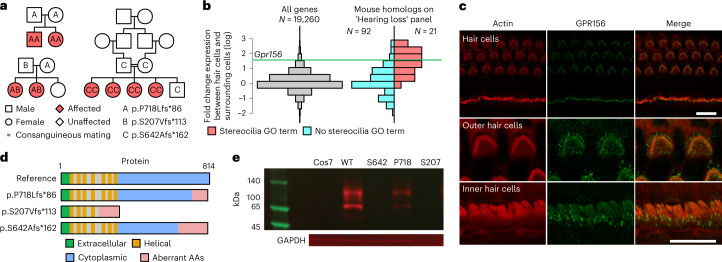
Loss-of-function variants in *GPR156* give rise to recessive congenital hearing loss. **a**, Schematic of the three pedigrees with cases homozygous or compound heterozygous for loss-of-function variants in the canonical transcript of *GPR156*, ENST00000464295.6. Blank symbols indicate individuals with an unknown genotype. **b**, Histograms of expression log fold changes for different sets of genes in mouse hair cells compared with surrounding cells: all mouse genes (left) and mouse genes homologous to their human counterparts in the ‘Hearing loss’ PanelApp panel, stratified by whether they had a stereocilia-related Gene Ontology (GO) term (that is, a term whose name contained ‘stereocilia’ or ‘stereocilium’ or the descendant of such a term) (right). The log fold change for *Gpr156* is shown as a horizontal line. **c**, Maximum intensity projections of confocal Z stacks in the organ of Corti and vestibular system of a P10 wild-type mouse immunostained with GPR156 antibody (green) and counterstained with phalloidin (red). Top row, overview of the organ of Corti and vestibular system. Middle and bottom rows, magnified images of outer hair cells and inner hair cells, respectively. No stereociliary bundle staining was observed. The punctate staining observed in the organ of Corti was absent or significantly decreased in the utricle of the vestibular system. Scale bars, 10 μm (each image is representative of three replicates). **d**, Schematic showing the effects of each variant at the cDNA and amino acid level and on the protein product. **e**, Exemplar western blot taken from three replicates of GPR156–GFP using anti-GPR156 antibody in untransfected Cos7 cells (Cos7); Cos7 cells transfected with the wild-type construct (WT); and Cos7 cells transfected with the constructs containing each of the mutant alleles p.S642Afs*162 (S642), p.P718Lfs*86 (P718) and p.S207Vfs*113 (S207). Source data

**Extended Data Fig. 9:**
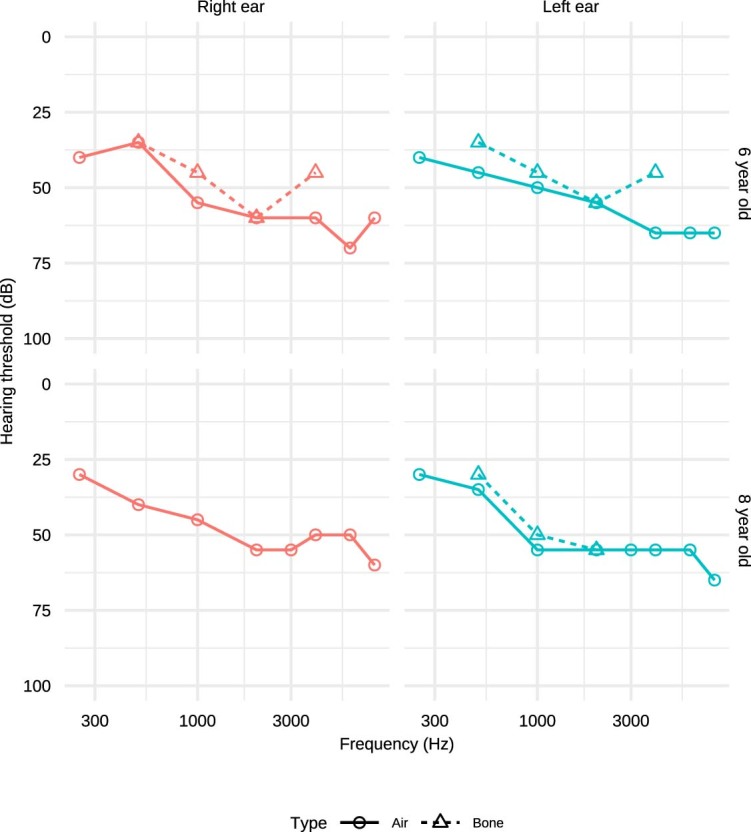
Illustrative audiograms for *GPR156* cases. Air and bone conduction audiograms for the two affected daughters of the family with compound heterozygous *GPR156* truncating alleles.

BeviMed identified a recessive genetic association between high-impact variants in *GPR156* and the Specific Disease ‘Congenital hearing impairment’. Two high-impact variants in *GPR156* were responsible for the strong evidence of association: a 1-bp insertion predicting p.S207Vfs*113 and a 1-bp insertion predicting p.P718Lfs*86 with respect to the canonical Ensembl transcript ENST00000464295.6. One family contained two affected siblings who were both homozygous for the p.S207Vfs*113 variant inherited from heterozygous parents. In a second family, there were also two affected siblings, in this case compound heterozygous for the same p.S207Vfs*113 variant that was maternally inherited and a different p.P718Lfs*86 variant that was paternal. Using GeneMatcher^31^, we identified a third pedigree from Saudi Arabia with biallelic truncating variants in *GPR156*. This consanguineous pedigree contained four siblings with hearing impairment, all of whom were homozygous for a variant predicting p.S642Afs*162 (Fig. 4a). The eight affected individuals in these three families all had congenital nonsyndromic bilateral sensorineural hearing loss (see Extended Data Fig. 9 for illustrative audiograms).

*GPR156* encodes probable G protein-coupled receptor 156, which has sequence homology to the class C GABAB receptors^32^. Although previously designated as an orphan receptor, *GPR156* has recently been identified as a critical regulator of stereocilia orientation on hair cells of the auditory epithelium and other mechanosensory tissues^33^. Its expression is highly restricted to hair cells in the inner ear^34^. Disruption of stereocilia is a common pathogenic mechanism underlying many human Mendelian hearing loss disorders^35^, and the overexpression of *GPR156* in hair cells relative to surrounding cells was commensurate with the overexpression of the 21 genes currently implicated in hearing impairment having a Gene Ontology term relating to stereocilia (Fig. 4b). By immunostaining of the Corti and vestibular system from wild-type mice, we found that GPR156 strongly colocalizes with actin at the apical surface of the outer and inner hair cells of the organ of Corti (Fig. 4c).

The p.S207Vfs*113 variant is located in the sixth of 10 exons of *GPR156* and therefore, predicts absent expression through nonsense-mediated decay of the *GPR156* mRNA. In contrast, the p.S642Afs*162 and p.P718Lfs*86 variants both occur within the final *GPR156* exon and likely result in expression of abnormal GPR156 with an altered amino acid sequence and premature truncation of the cytoplasmic tail (Fig. 4d). To determine the effect of the variants on protein expression, we transfected Cos7 cells, which do not express *GPR156* endogenously, with constructs containing cDNAs for wild-type *GPR156* or *GPR156* containing each of the three mutant alleles, tagged with a green fluorescent protein (GFP) reporter. While cells transfected with wild-type sequence expressed GPR156–GFP fusion protein robustly, cells transfected with the mutant constructs either did not express the protein appreciably or exhibited markedly reduced expression, suggesting that all three of the truncated proteins are degraded (Fig. 4e). These data suggest that the biallelic chain truncating variants in *GPR156* cause a congenital hearing loss by preventing expression of GPR156 protein, thereby disrupting stereocilia formation in the auditory epithelium.

## Discussion

The standardization of GS within a health care system, together with powerful frameworks for genetic and phenotypic data processing and statistical analysis, promises to advance the resolution of the remaining unknown etiologies of rare diseases. We have developed a lightweight and easily deployable RDB, the Rareservoir, for genetic analysis of rare diseases using approaches such as BeviMed. In one unified analysis, we identified 260 associations, of which 241 had been published previously in a body of work spanning several decades of genetics research. Our results give an upper bound on the false discovery rate of 7.3%. In contrast, a recent analysis of 57,000 samples in the 100KGP reported 249 known and 579 previously unidentified associations^36^, giving an upper bound on the false discovery rate of 70%, which suggests that our analytical approach has a greater specificity for a given sensitivity. The associations spanned 86 disease classes across a wide range of organ systems. Interestingly, only 64% of the variants contributing substantially to the known associations were present in the table of clinically reported variants available at the time of this study. This suggests that, as cohorts grow larger, the results of statistical inference could help guide the clinical reporting process. The case sets we used in our genetic association analysis were based on the formal disease classifications used by the 100KGP. Some of the case sets, such as ‘Intellectual disability’ (5,529 probands), are particularly large and likely to be highly genetically heterogeneous, potentially limiting the power of our analyses. Careful partitioning of heterogeneous case sets using individual-level HPO terms^6^ has the potential to boost power. Of the 19 previously unidentified associations, we shortlisted, replicated and validated three. These three etiologies involve genes that had not previously been implicated in any of these human diseases. The remaining 16 associations include further plausible hypotheses. For example, *LRRC7*, which we identified to be associated with intellectual disability, encodes a brain-specific protein in postsynaptic densities^37^, and LRRC7-deficient mice exhibit a neurobehavioral phenotype^38^. *USP33*, which we found to be associated with early-onset hypertension, encodes a deubiquitinating enzyme implicated in regulating expression of the β2-adrenergic receptor regulation^39^. These and other candidates will require replication and validation before they can be considered causative genes.

The present study has several limitations. First, approximately 82% of the participants in the 100KGP are of European ancestry. While this percentage is in line with the proportion of residents in England and Wales reporting their ethnic group as white in the 2011 UK census (86%), its large magnitude constrains power to identify causative variants specific to other ancestry groups. Second, of the 269 case sets analyzed, 28 contained fewer than five probands, limiting power to identify the causes of the corresponding disease classes and highlighting the need for continued enrollment of patients with ultra-rare disorders. Third, we have only considered SNVs and indels in coding genes. The exploration of structural variants and of rare variation in noncoding genes and in regulatory elements of the genome may help identify further etiologies. Lastly, we focused our attention on monogenic models of rare disorders, even though the genetic etiologies of certain rare diseases may be polygenic. In addition, important variation in clinical presentation of monogenic disorders may be explained by polygenic effects. These limitations point toward multiple promising avenues of future research to uncover the remaining unknown genetic determinants of rare diseases.

## Methods

### Motivation for developing a sparse RDB

Computational approaches for discovering the etiologies of rare diseases typically depend on the analysis of a heterogeneous set of files, each of which can be very large and follow a distinct convention. Genotypes, for example, are ordinarily stored in VCFs containing data for one sample or for multiple samples. In the latter case, the data are usually distributed in files covering many different ‘chunks’ of the reference genome. Variant-level information, such as consequence predictions or pathogenicity scores, is typically encoded in strings that require extensive parsing to decode, either from within the VCFs containing the genotypes or in separate files. Modifying genotype or annotation files (for example, to incorporate newly generated data) requires rewriting files in their entirety. Phenotype data, pedigree data and the results of statistical inference are stored in a further set of files. Consequently, analyses are often burdensome to conduct and prone to error. Frameworks, such as Hail^7^ and OpenCGA^8^, afford greater flexibility, but they depend on the centrally organized deployment of a distributed storage system, hindering usability and portability.

RDBs are widely used, mature technologies, well known for their speed, reliability, flexibility, structure and extensibility. In the context of rare diseases, an RDB can in principle render the modification, combination and addition of data on samples, variants, genes and other entities efficient, reliable and straightforward to implement using a single query language. Unfortunately, the performance of RDBs degrades substantially when the number of records in a table reaches several billion, and the number of genotypes in a cohort the size of the 100KGP easily surpasses this threshold. However, the MAFs of pathogenic variants with strong effects on rare disease risk are typically kept below 1/1,000 by negative selection, and the proportion of nonhomozygous reference genotypes for variants within that MAF stratum is only about 1% of the total (Extended Data Fig. 1). Consequently, it is possible to construct a compact RDB that includes virtually all the pathogenic variants even in a large cohort such as the 100KGP. This provides an opportunity for exploiting the benefits of a single unified RDB containing the nonhomozygous genotypes of rare variants upon which to conduct the entirety of the etiological discovery process. Furthermore, it provides a natural foundation for developing web applications for the multidisciplinary review of genetic, phenotypic, statistical and other data.

### Rareservoir

The Rareservoir is an RDB schema and a complementary software package rsvr for working with rare disease data. The database stores data including rare variant genotypes, variant annotations, phenotypes, sample information and pedigrees (Extended Data Fig. 1), but it can be extended arbitrarily. A Rareservoir is built through a series of steps from a set of input data and parameters (Extended Data Fig. 3). The ‘bcftools’ program^47^ extracts (‘bcftools view’) and normalizes (‘bcftools norm’) variants from either a set of single-sample genome variant call format files (gVCFs) or from a merged VCF. In all steps of the procedure, variants are encoded as RSVR IDs using the ‘rsvr enc’ tool. Merged VCFs typically contain cohort-wide variant quality information in the FILTER column, which can be used to select variants for processing. However, this is not readily obtained from single gVCFs. To address this, we developed the ‘rsvr depth’ tool, which computes variant quality pass rates at all positions in the genome based on a random subsample of gVCFs. If the input is a merged VCF, an internal (that is, within-VCF) allele frequency threshold is applied with bcftools to filter out internally common variants. If the input is a set of single-sample gVCFs, internally common variants are filtered out in two steps, for computational efficiency. First, a set of variants that are statistically almost certain to be common based on a random sample of gVCFs is identified—by default, the variants for which a one-sided binomial test under the null hypothesis that the MAF = 0.01 is rejected at a significance level of 10^−6^ (done using the ‘rsvr tabulate’ tool). Second, all gVCFs are read sequentially, filtering out the variants identified in the previous step (using the ‘rsvr mix’ tool) and those for which the pass rates identified with ‘rsvr depth’ do not meet the threshold. Retained genotypes are then loaded into a temporary genotype table in the database in order to apply the final internal allele frequency filter by executing an SQL ‘DELETE’ statement. These variants are then annotated with gnomAD ‘probabilistic minor allele frequency’ (PMAF) scores^3^ using the ‘rsvr pmaf’ tool. The PMAF score is calculated with respect to a given allele frequency threshold *t* by evaluating a binomial test (at a significance threshold of 0.05) on the observed frequency of the variant under the null hypothesis that the variant has an allele frequency of *t*. If in any gnomAD population, the null is rejected for *t* = 0.001 and the allele count is at least two, the score is set to zero. If the null is rejected for *t* = 0.0001, the score is set to one. If the null is not rejected, the score is set to two. Finally, if the variant is absent from gnomAD, the score is set to three. For the nonpseudoautosomal dominant regions of chromosome X, only allele counts for males are used in calculations. Variants are then additionally annotated with their CADD phred scores using the ‘rsvr ann’ program and loaded into the VARIANT table. At this point, variants in the VARIANT and GENOTYPE table that have a PMAF score of zero may be deleted because they are unlikely to be involved in rare diseases. We then annotate the retained variants with predicted transcript consequences for a given set of transcripts specified in a Gene Transfer Format file. The 100KGP Rareservoir uses Ensembl v.104 canonical transcripts with a protein-coding biotype, of which >90% are MANE (Matched Annotation from National Center for Biotechnology Information (NCBI) and European Bioinformatics Institute (EBI))^48^ transcripts. The ‘rsvr seqfx’ program determines a set of SO terms for each interacting transcript–variant pair and encodes them as a CSQ ID, which is added to the CONSEQUENCE table. This table can also hold Loss-Of-Function Transcript Effect Estimator (LOFTEE) scores^10^ corresponding to a transcript–variant pair. Note that, as LOFTEE scores on the Genomics England Research Environment correspond to Ensembl v.99 transcripts, we mapped Ensembl v.104 canonical transcripts to the most similar v.99 transcripts having an identical coding sequence in order to obtain the LOFTEE scores for the 100KGP Rareservoir, finding a match for >98% of transcripts. The contents of the Gene Transfer Format file are also imported into the database to create tables of transcript features (FEATURE), transcripts (TX) and genes (GENE). Optionally, VARIANT, GENOTYPE and CONSEQUENCE may be filtered for RSVR IDs that have CSQ IDs meeting particular criteria: for instance, to retain only variants with protein-coding consequences. The SAMPLE table of metadata and genetic statistics for each sample represented in the input VCF(s) must then be added to the database, including mandatory columns containing the ID, sex, family and an indicator of inclusion in the maximal unrelated set of samples in the database. The VARIANT, GENOTYPE and CONSEQUENCE tables are indexed by RSVR ID to support fast lookups by genomic location. The SAMPLE table and GENOTYPE table are indexed by sample ID, allowing fast lookups by sample. The CONSEQUENCE, TX and GENE tables are indexed by transcript and gene ID, allowing fast lookups of variants based on gene/transcript-specific consequences. If sample phenotypes have been encoded using phenotypic terms (for example, International Classification of Diseases 10 (ICD10) codes or HPO terms), terms from the relevant coding systems can be added to a generic PHENOTYPE table mapping code IDs to descriptions, and codes assigned to samples can be added to the SAMPLE_PHENOTYPE table. Disease labels may be added to the CASE_SET table. The majority of the compute time required for building the database is taken by reading the genotype data from the input VCFs, which may be executed in parallel over separate regions against a merged VCF or over single gVCFs. The rsvr tool, implemented in C++, executes rapidly, with ‘rsvr seqfx’ capable of assigning CSQ IDs for all Ensembl v.104 canonical transcripts to all variants (over 685 M) in gnomAD v.3.0 in under 40 min on a single core. The 100KGP Rareservoir, which is stored in an SQLite database, returns complex gene-specific queries in under 1 s. For example, (1) a table with 628 rows containing the moderate- and high-impact variants with a PMAF score ≥1 in *TTN* along with the corresponding consequence predictions and CADD scores takes 0.57 s; (2) a table with 1,498 rows containing, for each variant, the samples and genotypes for individuals who carry an alternate allele takes 0.61 s; and (3) a classification for each of the 77,539 participants into proband with the Specific Disease ‘Dilated cardiomyopathy’, relative of such a proband, unrelated control, or relative of a control takes 0.65 s. Specific details on implementation of the workflow, code for encoding data as SQL statements compatible with Rareservoir and the mapping between bits in the 64-bit CSQ ID and each SO term assigned by rsvr seqfx can be found in the rsvr software package (see the code availability). Software packages rsvr 1.0, bcftools 1.9 and perl 5 were used to build the 100KGP Rareservoir.

### Encoding RSVR IDs

SNVs and indels may be encoded as 64-bit integers called RSVR IDs. To compute an RSVR ID for a given variant, the following expression is evaluated:

$$c \times 2^{58} + p \times 2^{30} + \left| r \right| \times 2^{24} + \left| a \right| \times 2^{18} + \mathop {\sum}\limits_{{\mathrm{i}} = 1}^{\left| A \right|} {A_{\mathrm{i}} \times 4^{{\mathrm{i}} - 1}} ,$$

where *c* is the chromosome number (using 23, 24 and 25, respectively, to represent chromosomes X, Y and MT); *p* is the position; and |*r*| and |*a*| are the lengths of the reference and alternate alleles, respectively. *A* is a sequence identical to the alternate allele, *a*, when its length is less than 10 and otherwise, equal to the first five followed by the last four elements of *a*. In the summation, nucleotides are assigned values A = 0, C = 1, G = 2 and T = 3. The expression evaluates to integers that can be represented using 63 bits, setting the most significant bit to zero when encoding as 64-bit integers. The chromosome, position, reference, alternate allele lengths and alternate allele bases are thereby encoded, respectively, by the subsequent 5, 28, 6, 6 and 18 bits (with 2 bits per base for the alternate allele). This procedure and its inverse are implemented in the ‘rsvr enc’ and ‘rsvr dec’ programs, respectively. The reference and alternate alleles of input variants are first normalized by removing any redundant identical sequence from the starts and then, the ends. The proportion of variants in gnomAD 3.0 weighted by allele count that can be encoded losslessly is 99.3%, while 99.8% can be represented by a distinct RSVR ID. The full variant information corresponding to any encountered ambiguous RSVR ID may be stored in full in an additional table. Structural variants that can be represented by a position and length may also be encoded using distinct 64-bit RSVR IDs alongside SNVs and indels by setting the most significant bit to one and subsequently, encoding the type of structural variant using 2 bits (deletion 0, duplication 1, inversion 2, insertion 3), the chromosome using 5 bits (as done for SNVs and indels), and the start and length consecutively using 28 bits.

### Genetic association analysis of 100KGP data

We constructed a Rareservoir in the Genomics England Research Environment containing the PASSing^49^ variants in the merged VCF of 77,539 consented participants in the 100KGP Rare Diseases Programme. This Rareservoir only included variants with a PMAF > 0 according to GnomAD v.3.0, an internal MAF < 0.002 and at least one predicted consequence on a canonical transcript in Ensembl v.104. Variants with a greater MAF are unlikely to be highly penetrant for diseases eligible for inclusion in the 100KGP and are likely to have, at most, small effects on risk, making them challenging to validate. Variants with a median genotype quality <35 and SNVs with a CADD Phred score <10 were also excluded from the analyses.

For each of the 269 rare disease classes (Extended Data Figs. 5 and 6), we applied the BeviMed^9^ association test to rare variants extracted from the Rareservoir database in each of the 19,663 canonical transcripts belonging to a gene with a ‘protein_coding’ biotype. The analysis was carried out using R 3.6.2, making use of functionality from packages Matrix 1.2–18, dplyr 0.8.5, bit64 0.9–7, bit 1.1–14, DBI 1.1.0, RSQLite 2.1.4 and BeviMed 5.7. The case set for a given disease class and gene was constructed by selecting one case from each pedigree containing at least one person affected with the disease class. For the purposes of the association analysis, participants were labeled ‘explained’ by a given gene if they had variants in that gene classified as ‘pathogenic_variant’ or ‘likely_pathogenic_variant’ in the ‘gmc_exit_questionnaire’ table in the Genomics England Research Environment. To boost power, we used this information to reassign cases that were explained by variants in a different gene to the control group.

Using BeviMed, we performed a Bayesian comparison of a baseline model of no association and each of six association models defined by an MOI and a class of etiological variant.

1. No association (prior probability 0.99)
2. Dominant association with ‘high’-impact variants having a PMAF ≥ 2 (that is, corresponding to a target MAF < 0.01%; prior probability 0.002475)
3. Dominant association with ‘moderate’-impact variants having a PMAF ≥ 2 (prior probability 0.002475)
4. Dominant association with ‘5′ UTR’ variants having a PMAF ≥ 2 (prior probability 0.00005)
5. Recessive association with ‘high’-impact variants having a PMAF ≥ 1 (that is, corresponding to a target MAF < 0.1%; prior probability 0.002475)
6. Recessive association with ‘moderate’-impact variants having a PMAF ≥ 1 (prior probability 0.002475)
7. Recessive association with 5′ UTR variants having a PMAF ≥ 1 (prior probability 0.00005)

Thus, the overall prior probability of association was 0.01, and there was an equal prior probability of dominant and recessive inheritance. The PPA was the sum of the posterior probabilities of models 2–7. We imposed a stricter PMAF threshold under a dominant MOI than under a recessive MOI because ceteris paribus, dominant variants are under stronger negative selection than recessive variants. The three groups of variants were selected as follows.

- 5′ UTR variants: those with a 5_prime_UTR_variant consequence
- High-impact variants: those with any consequence amongst start_lost, stop_lost, frameshift_variant, stop_gained, splice_donor_variant or splice_acceptor_variant, excluding variants with a ‘low-confidence’ LOFTEE score^10^
- Moderate-impact variants: those with any consequence amongst start_lost, stop_lost, frameshift_variant, stop_gained, splice_donor_variant or splice_acceptor_variant, missense_variant or inframe_deletion

The rationale for embedding variants from the high-impact class in the moderate-impact class is that both types of variant are capable of inducing a loss of function. The prior on the probability that a modeled rare variant is pathogenic, conditional on either the association model mediated by 5′ UTR variants or the association model mediated by moderate-impact variants, was set to Beta(2,8). This encodes a prior conditional expectation that 20% of rare variants are pathogenic, which is well suited to missense and 5′ UTR variants. However, we specified a distribution with a greater mean for the high-impact models. Specifically, the prior on the probability that a modeled high-impact variant is pathogenic was set to Beta(3,1), which reflects a prior conditional expectation that 75% of rare variants are pathogenic because loss-of-function variants tend to be functionally equivalent to each other. BeviMed reports the posterior probability that each variant is pathogenic conditional on the MOI and the class of etiological variant. The methodology is described in further detail in the original BeviMed publication^9^.

We applied the following postprocessing of BeviMed results with a PPA > 0.95.

- We reran BeviMed including all samples (that is, with relatives of cases and controls). Associations for which the analysis with all samples caused the PPA to fall below 0.9 were filtered out due to conflicting evidence for the association within families.
- We reran BeviMed after removing variants absent from affected relatives of the cases. Associations for which this removal caused the PPA to drop below 0.25 were filtered out because they depended on variants that were not shared by affected cases within families.
- To guard against false positives due to incorrect pedigree data, population structure or cryptic relatedness, we applied the following algorithm. We obtained the distribution of the number of rare variants in the Rareservoir shared by pairs of individuals within each assigned ancestry in the 100KGP. The top percentile in each of these distributions was used to indicate potential relatedness between participants in the same population. We reran BeviMed after removing cases so as to ensure that no more than one case from any set of potentially related cases sharing a variant was included in the analysis. Associations for which this analysis caused the PPA to fall below 0.25 were filtered out.

To account for correlation between case sets, for each gene, we removed all but the most strongly associated disease class within each disease group before reporting the 260 associations remaining. Without the postprocessing, the number of reported associations would have been 302. Conditional on the modal model underlying each of the 260 associations, we recorded the variants with a posterior probability of pathogenicity >0.8 accounting for at least one case in the 100KGP.

### PanelApp annotation

Significant associations were colored according to PanelApp^14^ (Fig. 1b) evidence levels for panel–gene relations (green for high evidence, amber for moderate evidence and red for low evidence) for panels of type ‘Rare Disease 100K’, which are organized hierarchically by Disease Sub Group and Disease Group, or of type ‘GMS Rare Disease’. Given an association between a gene and a case set (corresponding either to a Specific Disease or to a Disease Sub Group), we searched for panels that contained the gene and had the same name as the case set (ignoring case). If such a match was not found, we searched for panels that contained the gene and that belonged to a Disease Sub Group with the same name as the Disease Sub Group of the case set. When this matching rule generated multiple matches, we selected the panel(s) with the highest evidence. If multiple panels still remained, we selected the panel with the smallest number of genes. Associations for which no matching panel in PanelApp could be found were inspected manually to assess whether PanelApp contained an alternative suitable panel (marked with an asterisk in Fig. 1b).

### Shortlisting previously unidentified genetic associations for validation

Several sources of independent evidence were used to shortlist significant associations for validation. For each source, a score of one was awarded if the evidence was supportive and zero otherwise. Scores were then added over the different sources and used to rank the associations. Associations for which at least three sources of evidence were supportive were taken forward for further investigation. The sources of evidence and qualifying criteria for being considered supportive are listed below. Note that here we refer to variants that had a probability of pathogenicity >0.8 conditional on the modal model as ‘probably pathogenic’.

- Counting cosegregating pedigree members. The pedigrees harboring pathogenic configurations of probably pathogenic alleles were checked for cosegregation between genotype and affection status. This evidence counted as supportive for associations for which all such pedigrees demonstrated cosegregation and there were at least three additional relatives who had not been included in the association analysis but for whom there was cosegregation. Note that Binary Alignment and Map (BAM) files for the affected members of pedigrees who were called homozygous reference for probably pathogenic variants were checked for evidence of mosaicism to guard against the possibility that they were falsely portraying a lack of cosegregation.
- pLI and *Z* scores. pLI and *Z* scores for depletion of missense variants were obtained from the gnomAD v.2.2.1 browser^10^. pLI > 0.9 for associations in which high-impact variants were most strongly associated was counted as supportive, whilst *Z* scores >2 for associations in which moderate-impact variants were most strongly associated were counted as supportive.
- Recessive association. Population genetic metrics of purifying selection (pLI scores and *Z* scores) are sensitive to depletion of high-impact variants and missense variants, respectively. They are, therefore, useful measures to corroborate dominant associations. However, these metrics have low sensitivity to identify the signatures of selection against recessive diseases because isolated pathogenic variants in heterozygous form do not lead to a reduction in reproductive fitness. To avoid disadvantaging recessive associations identified by BeviMed, they were assigned a contribution of one point to the score.
- Literature review. A comprehensive literature review assessing the gene’s role (if any) in biological processes relevant to the disease, other diseases and a survey of model organisms was undertaken and determined to be either supportive or not.

### *ERG*: primary endothelial cell culture

Single-donor primary HDLECs (Promocell) were cultured in Endothelial Cell Growth Medium MV2 (Promocell). Pooled donor HUVECs (Lonza) were grown in Endothelial Cell Growth Media-2 (Lonza). HUVECs and HDLECs were grown on 1% (vol/vol) gelatin and used between passages 3 and 5.

### *ERG*: real-time PCR

HUVECs and HDLECs were grown to confluency in a pregelatinized six-well dish. Total RNA was isolated using the RNeasy Mini Kit (Qiagen), and 1 µg of total RNA was transcribed into cDNA using Superscript III Reverse Transcriptase (Thermo Fisher Scientific). Quantitative real-time PCR was performed using PerfCTa SYBR Green FastMix (Quanta Biosciences) on a Bio-Rad CFX96 System. Gene expression values of ERG in HUVECs and HDLECs were normalized to GAPDH expression and compared using the ΔΔC_T_ method. The following oligonucleotides were used: ERG, 5′-GGAGTGGGCGGTGAAAGA-3′ and 5′-AAGGATGTCGGCGTTGTAGC-3′; GAPDH, 5′-CAAGGTCATCCATGACAACTTTG-3′ and 5′-GGGCCATCCACAGTCTTCTG-3′.

### *ERG*: immunoblotting analysis

Immunoblotting was performed according to standard conditions. Proteins were labeled with the following primary antibodies: rabbit anti-human ERG antibody (1:1,000; ab133264; Abcam) and mouse anti-human GAPDH (1:10,000; MAB374; Millipore). Primary antibodies were detected using fluorescently labeled secondary antibodies: goat anti-rabbit IgG DyLight 680 and goat anti-mouse IgG Dylight 800 (Thermo Scientific). Detection of fluorescence intensity was performed using an Odyssey CLx imaging system (Li-COR Biosciences, Lincoln) and Odyssey v.4 software.

### *ERG*: immunofluorescence analysis of endothelial cells and mouse tissues

Confluent cultures of HUVECs and HDLECs were fixed with 4% (wt/vol) paraformaldehyde for 15 min and permeabilized with 0.5% (vol/vol) Triton X-100 before incubation with 3% BSA (wt/vol) in phosphate buffered saline (PBS) containing the following primary antibodies: goat anti-human PROX1 antibody (1:100; AF2727; R&D Systems), rabbit anti-human ERG antibody (1:100; ab92513; Abcam) and mouse anti-human VE-cadherin (1:100; 555661; BD Biosciences). Secondary antibody incubation was carried out in 3% BSA (wt/vol) in PBS using the following antibodies: donkey anti-goat IgG Alexa Fluor-488 (1:1,000; A-11055), donkey anti-rabbit IgG Alexa Fluor-555 (1:1,000; A-31572) and donkey anti-mouse Alexa Fluor-594 (1:1,000; A-21203). All secondary antibodies were from Thermo Fisher Scientific. Nuclei were visualized using DAPI (Sigma-Aldrich). Confocal microscopy was carried out on a Carl Zeiss LSM 780 confocal laser scanning microscope with Zen 3.2 software. All animal experiments were conducted with ethical approval from Imperial College London under UK Home Office Project Licence PEDBB1586 in compliance with the UK Animals (Scientific Procedures) Act of 1986. Ear tissue was collected from euthanized 3-week-old male and female C57BL/6J mice and fixed in 4% (wt/vol) paraformaldehyde at room temperature for 2 h. Tissue was then washed with PBS followed by a blocking and permeabilization step using 3% (wt/vol) milk in phosphate-buffered saline solution containing 0.3% (vol/vol) Triton X-100 (PBST) for 1 h at room temperature. The following primary antibodies were used for immunofluorescence staining: goat anti-human PROX1 antibody (1:100; AF2727; R&D Systems) and rabbit anti-human ERG antibody (1:100; ab92513; Abcam). Primary antibodies were incubated at 4 °C overnight in 3% (wt/vol) milk in PBST. The following day, tissues were washed three times with PBST over the course of 2 h at room temperature. Tissues were incubated with secondary antibodies at room temperature for 2 h in 3% milk (wt/vol) in PBST. Primary antibodies were detected using fluorescently labeled secondary antibodies: donkey anti-goat IgG Alexa Fluor-488 (1:400; A-11055; Thermo Fisher Scientific) and donkey anti-rabbit IgG Alexa Fluor-555 (A-31572; Thermo Fisher Scientific). Stained samples were mounted onto glass slides using Fluoromount G (Thermo Fisher Scientific). Images were acquired using a Zeiss LSM 780 confocal laser scanning microscope with Zen v.3.2 software. All confocal images represent maximum intensity projection of Z stacks of single tiles.

### *ERG*: subcloning and overexpression in HEK293 cells

We subcloned *ERG* (ENST00000288319.12) from HUVECs into the mammalian expression vector pcDNA3.1 (Thermo Fisher). *ERG* variants were generated by site-directed mutagenesis using the Quikchange Lightning kit (Agilent) using the wild-type *ERG* cDNA as the template. Expression of wild-type and mutant *ERG* was carried out using polyethylenimine (Sigma-Aldrich) transfection reagent in HEK293 cells grown in Dulbecco’s Modified Eagle Medium (DMEM) (Thermo Fisher) with 10% (vol/vol) FBS. After 24 h, cells were fixed with 4% (wt/vol) paraformaldehyde for 15 min and permeabilized with 0.5% (vol/vol) Triton X-100 before incubation with 3% BSA (wt/vol) in PBS containing mouse monoclonal anti-ERG antibody (1:100; sc-376293; Santa Cruz Biotechnology). Secondary antibody incubation was carried out in 3% BSA (wt/vol) in PBS using donkey anti-mouse Alexa Fluor-488 (1:1,000; A-21202; Thermo Fisher). Nuclei were visualized using DAPI (Sigma-Aldrich). Confocal microscopy was carried out on a Carl Zeiss LSM780 confocal laser scanning microscope with Zen 3.2 software.

### *ERG*: estimation of nuclear and nonnuclear ERG in HEK293 cells

Each image was read into a pair of channel-specific 1,024 × 1,024 matrices in R v.4.2.1 using the readCzi function from the readCzi R package v.0.2.0. A pixel was declared to contain a nuclear region if the intensity in the blue channel exceeded 60% of the 95th percentile of blue intensities across all pixels above background (identified as exceeding 1.35 × 10^−2^ by visual inspection of bimodal intensity histograms). A pixel was declared to contain ERG if the intensity in the green channel exceeded 30% of the 95th percentile of the green intensities within the pixels previously declared to be nuclear. To fill in intranuclear gaps, any nonnuclear pixels adjacent to at least five nuclear pixels were declared nuclear. The estimated proportion of ERG that was cytosolic in an image was set to the number of ERG pixels that did not overlap nuclear pixels divided by the number of ERG pixels.

### *GPR156*: western blots

We subcloned *GPR156* from human brain cDNA into EGFP-N2 vector. The three mutant *GPR156* constructs were generated by mutagenesis using the QuickChange kit (Stratagene) and wild-type GPR156–GFP as a template. For expression analysis, the wild type and mutant constructs were transfected in COS7 cells grown in DMEM (Gibco) with 10% FBS. Transfections were performed with Lipofectamine 2000 reagent (Life Technologies). Cells were harvested 48 h after transfection; lysed in buffer containing 1% 3-[(3-cholamidopropyl)dimethylammonio]-1-propane sulfonate (CHAPS), 100 mM NaCl and 25 mM N-2-hydroxyethylpiperazine-N-2-ethane sulfonic acid (HEPES), pH 7.4; and clarified by centrifugation at 18,407*g*. Lysates (20 μg) were run on a 4–20% sodium dodecyl sulfate–polyacrylamide gel electrophoresis (SDS-PAGE) gel. The membrane was blocked with 5% milk, incubated with anti-GPR156 (1:200) and developed with horseradish peroxidase (HRP)-conjugated secondary (sheep anti-rabbit) antibody (1:1,000). Comparable loading was checked by stripping and reprobing the blots with anti-GAPDH (1:500) antibody (Santa Cruz Biotechnology).

### *GPR156*: whole-mount immunostaining of GPR156 in mouse inner ears

All the animal work was approved by the University of Maryland, Baltimore Institutional Animal Care and use Committee (IACUC 420002). Inner ears were dissected from C57BL/6J mice with a postnatal age of 10 days and fixed in 4% paraformaldehyde in PBS overnight. For whole-mount immunostaining, the cochleae were microdissected and were subjected to blocking for 1 h with 10% normal goat serum in PBS containing 0.25% Triton X-100, followed by overnight incubation at 4 °C with anti-GPR156 antibody (1:200; PA5-23857; Thermo Fisher) in 3% normal goat serum with PBS. F-actin was decorated using phalloidin (1:300). Confocal images were acquired from a Zeiss LSM710 confocal microscope, and images were processed using ImageJ v.1.53t software.

### Ethics

The 100,000 Genomes project was approved by East of England–Cambridge Central Research Ethics Committee ref:20/EE/0035. Only participants who provided written informed consent for their data to be used for research were included in the analyses. The study at the University of Maryland was approved by the institutional review board (RAC no. 2100001), and written informed consent was obtained by clinicians at King Faisal Hospital in Saudi Arabia from the participating individuals. The study of the Japanese ancestry pedigrees bearing *PMEPA1* truncating alleles was approved by the Institutional Review Board of the National Cerebral and Cardiovascular Centre (M14-020) and Sakakibara Heart Institute (16–035), and written informed consent was obtained from the participating individuals.

### Reporting summary

Further information on research design is available in the Nature Portfolio Reporting Summary linked to this article.

## Online content

Any methods, additional references, Nature Portfolio reporting summaries, source data, extended data, supplementary information, acknowledgements, peer review information; details of author contributions and competing interests; and statements of data and code availability are available at 10.1038/s41591-023-02211-z.

## Supplementary information

Reporting Summary

## Data Availability

Genetic and phenotypic data for the 100KGP study participants are available through the Genomics England Research Environment via the application at https://www.genomicsengland.co.uk/join-a-gecip-domain. PanelApp gene panels and evidence of associations were obtained using the PanelApp application programming interface (https://panelapp.genomicsengland.co.uk/api/docs/) on the 20 October 2021. CADD v.1.5 (https://cadd.gs.washington.edu/), gnomAD v.3.0 (https://gnomad.broadinstitute.org/) and Ensembl v.104 (http://may2021.archive.ensembl.org/index.html) were used for variant annotation. Source data are provided with this paper.

## Source data

Source Data Fig. 1 Sheet 1 shows a table of associations shown in Fig. 1b annotated with BeviMed PPAs (PPA), the level of the case set in the disease label hierarchy (Level), the inferred variant class and MOI for the association, the matched PanelApp panel for the association, the method that was used to find the match (Match method, either ‘Automatic’ or ‘Manual’), the associated evidence level for the match and the notes on the consistency between the MOI listed by PanelApp for the association and the inferred MOI (MOI match comment). Sheet 2 shows a table of variants having a probability of pathogenicity >0.8 conditional on the modal model and forming a pathogenic configuration of alleles in at least one case. While these variants contributed to the reported statistical associations, they have not been individually scrutinized according to ACMG guidelines.
Source Data Fig. 2 Uncropped western blot images corresponding to Fig. 2e.
Source Data Fig. 4 Uncropped western blot images corresponding to Fig. 4e.

## Code availability

The rsvr tool and Rareservoir schema are available from https://github.com/turrogroup/rsvr.
