# Supplementary material for: Genetic association analysis of 77,539 genomes reveals rare disease etiologies
Source: Nat Med. 2023 Mar 16;29(3):679–88. doi: 10.1038/s41591-023-02211-z (PMC10033407; doi:10.1038/s41591-023-02211-z)
Supplement: Supplementary file 1 — Reporting Summary [file 41591_2023_2211_MOESM1_ESM.pdf]

nature portfolio

Corresponding author(s): Ernest Turro

Last updated by author(s): Dec 23, 2022

Reporting Summary

Nature Portfolio wishes to improve the reproducibility of the work that we publish. This form provides structure for consistency and transparency in reporting. For further information on Nature Portfolio policies, see our Editorial Policies and the Editorial Policy Checklist.

Statistics

For all statistical analyses, confirm that the following items are present in the figure legend, table legend, main text, or Methods section.

n/a	Confirmed	
☐	☒	The exact sample size (*n*) for each experimental group/condition, given as a discrete number and unit of measurement
☐	☒	A statement on whether measurements were taken from distinct samples or whether the same sample was measured repeatedly
☐	☒	The statistical test(s) used AND whether they are one- or two-sided<br>*Only common tests should be described solely by name; describe more complex techniques in the Methods section.*
☐	☒	A description of all covariates tested
☐	☒	A description of any assumptions or corrections, such as tests of normality and adjustment for multiple comparisons
☐	☒	A full description of the statistical parameters including central tendency (e.g. means) or other basic estimates (e.g. regression coefficient) AND variation (e.g. standard deviation) or associated estimates of uncertainty (e.g. confidence intervals)
☐	☒	For null hypothesis testing, the test statistic (e.g. *F*, *t*, *r*) with confidence intervals, effect sizes, degrees of freedom and *P* value noted<br>*Give P values as exact values whenever suitable.*
☐	☒	For Bayesian analysis, information on the choice of priors and Markov chain Monte Carlo settings
☒	☐	For hierarchical and complex designs, identification of the appropriate level for tests and full reporting of outcomes
☒	☐	Estimates of effect sizes (e.g. Cohen's *d*, Pearson's *r*), indicating how they were calculated

Our web collection on statistics for biologists contains articles on many of the points above.

Software and code

Policy information about availability of computer code

Data collection	bcftools 1.9; perl 5.
Data analysis	rsvr 1.0; R 3.6.2 (packages: Matrix 1.2-18, dplyr 0.8.5, bit64 0.9-7, bit 1.1-14, DBI 1.1.0, RSQLite 2.1.4, BeviMed 5.7, readCzi 0.2.0); Odyssey 4, Zen 3.2, ImageJ 1.53t.

For manuscripts utilizing custom algorithms or software that are central to the research but not yet described in published literature, software must be made available to editors and reviewers. We strongly encourage code deposition in a community repository (e.g. GitHub). See the Nature Portfolio guidelines for submitting code & software for further information.

Data

Policy information about availability of data

All manuscripts must include a data availability statement. This statement should provide the following information, where applicable:

- Accession codes, unique identifiers, or web links for publicly available datasets
- A description of any restrictions on data availability
- For clinical datasets or third party data, please ensure that the statement adheres to our policy

Genetic and phenotypic data for the 100KGP study participants are available through the Genomics England Research Environment via application at https://www.genomicsengland.co.uk/join-a-gecip-domain. PanelApp gene panels and evidence of associations were obtained using the PanelApp application programming

interface (https://panelapp.genomicsengland.co.uk/api/docs/) on the 20th October 2021. CADD v1.5 (https://cadd.gs.washington.edu/), gnomAD v3.0 (https://cadd.gs.washington.edu/) and Ensembl v104 (http://may2021.archive.ensembl.org/index.html) were used for variant annotation.

Human research participants

Policy information about studies involving human research participants and Sex and Gender in Research.

Reporting on sex and gender	Breakdown by genetically determined sex for the Genomics England discovery cohort as provided in the Research Environment: 40,332 female; 35,511 male; 1,696 not available.
Population characteristics	Cohort of rare disease cases covering a wide range of pathlogies, as described in Extended Data fig. 5. Breakdown by genetically determined most probably ancestry for the genomics England discovery chort as provided in the Research Environment: African:2,762; Admixed American: 3,006; East Asian 573; European: 63,493; South Asian: 7,705. Ages of participants ranged between 0 and 110, with a lower quartile of 27, a median of 42 and an upper quartile of 58, with 18.4% under 18 overall.
Recruitment	Cases were recruited by referring clinicians through the National Health Service.
Ethics oversight	East of England–Cambridge Central REC REF 20/EE/0035. University of Maryland IRB (RAC#2100001), IRBs of the National Cerebral and Cardiovascular Centre (M14-020) and Sakakibara Heart Institute (16-035).

Note that full information on the approval of the study protocol must also be provided in the manuscript.

Field-specific reporting

Please select the one below that is the best fit for your research. If you are not sure, read the appropriate sections before making your selection.

☒ Life sciences ☐ Behavioural & social sciences ☐ Ecological, evolutionary & environmental sciences

For a reference copy of the document with all sections, see nature.com/documents/nr-reporting-summary-flat.pdf

Life sciences study design

All studies must disclose on these points even when the disclosure is negative.

Sample size	Statistical power to identify genetic associations with rare diseases depends on various factors including the sample sizes and genetic homogeneities of case groups. To our knowledge, a formal sample size calculation was not performed for the 100KGP. However, the study was informed by previous smaller studies showing sufficient power (see references in Turro et al. (2020), Nature). A small number of unrelated probands with a shared etiology can, in some cases, provide sufficient power to identify a true association (see Greene et al. (2017), American Journal of Human Genetics). Almost 90% of the case sets (distinct Sub Groups or Specific Diseases) contain 5 or more probands, providing power for detection for many of the 260 diseases classes under a wide range of conditions.
Data exclusions	None.
Replication	The 100KGP cohort is unique in its characteristics and scale, so it was not possible to reproduce the results of statistical association outright. However, validation was sought by analytical and experimental means, including searching for additional pedigrees in other collections and through bioinformatic and experimental follow up work.
Randomization	Recruitment and GS were performed concurrently across rare disease categories, thus randomising the order in which individuals were sequenced with respect to phenotype.
Blinding	This is an observational genetic study, not a clinical trial. As genome sequencing followed enrolment, participants and investigators were unaware of the participant genotypes generated by the 100KGP at enrolment.

Reporting for specific materials, systems and methods

We require information from authors about some types of materials, experimental systems and methods used in many studies. Here, indicate whether each material, system or method listed is relevant to your study. If you are not sure if a list item applies to your research, read the appropriate section before selecting a response.

Materials & experimental systems

n/a	Involved in the study
☐	☒ Antibodies
☐	☒ Eukaryotic cell lines
☒	☐ Palaeontology and archaeology
☐	☒ Animals and other organisms
☐	☒ Clinical data
☒	☐ Dual use research of concern

Methods

n/a	Involved in the study
☒	☐ ChIP-seq
☒	☐ Flow cytometry
☒	☐ MRI-based neuroimaging

Antibodies

Antibodies used

HRP conjugated secondary (sheep anti-rabbit) antibody: Cat#NA934V; 1:1000 Amersham ECL kit; goat anti-human PROX1 antibody (1:100; AF2727, R&D Systems); rabbit anti-human ERG antibody (1:100; ab92513, Abcam); mouse anti-human VE-cadherin (1:100; 555661, BD Biosciences); donkey anti-goat IgG Alexa Fluor-488 (1:1000; A-11055, Thermo Fisher); donkey anti-rabbit IgG Alexa Fluor-555 (1:1000; A-31572, Thermo Fisher); donkey anti-mouse Alexa Fluor-594 (1:1000; A-21203, Thermo Fisher); rabbit anti-human ERG antibody (1:1000; ab133264, Abcam); mouse anti-human GAPDH (1:500; MAB374, Millipore); goat anti-rabbit IgG DyLight 680 (Cat#35568; 1:10000, Thermo Scientific) and goat anti-mouse IgG Dylight 800 (Cat#SA5-10176; 1:10000, Thermo Scientific).

Validation

Goat anti-human PROX1 (AF2727): https://www.rndsystems.com/products/human-prox1-antibody_af2727?gclid=CjwKCAiAnZCdBhBmEiwA8nDQxZE5sZtYeoXEiB1O4huLE5EnUHQ-6Of9Z2MSTkoKLsqrxRzIBE5c2BoCFYIQAvD_BwE&gclsrc=aw.ds
Mouse anti-human VE-cadherin (555661): https://www.bdbiosciences.com/en-us/products/reagents/flow-cytometry-reagents/research-reagents/single-color-antibodies-ruo/purified-mouse-anti-human-cd144.555661.
Validation of Anti-ERG antibody has been demonstrated in multiple studies by the authors. For example, see Nat Commun. (2017);8:16002. doi: 10.1038/ncomms16002.
Validation data of GPR antibodies is given on manufacturer's website (sigma) and we further confirm it using GFP tagged full length protein both.

Eukaryotic cell lines

Policy information about cell lines and Sex and Gender in Research

Cell line source(s)

Cos7 cells lines are from ATCC. Single donor primary human dermal lymphatic endothelial cells (HDLEC) were from Promocell, Heidelberg, Germany. Pooled donor human umbilical vein endothelial cells (HUVEC) were from Lonza, Slough, UK. HEK-293 cells were from Takara Bio Inc., USA.

Authentication

HDLEC were assessed by the supplier (Promocell) using flow cytometry for CD31 (99% positive) and podoplanin (96% cells are positive).

Mycoplasma contamination

Promocell state that all HDLEC have been tested for the absence of HIV-1, HBV, HCV, and microbial contaminants (fungi, bacteria, and mycoplasma). Lonza state that all HUVEC test negative for mycoplasma, bacteria, yeast, and fungi. HIV-1, hepatitis B and hepatitis C are not detected for all donors and/or cell lots. The HEK-293 cells tested negative for mycoplasma. Cos7 cells tested negative for mycoplasma.

Commonly misidentified lines
(See ICLAC register)

HEK-293 cells have not been listed as commonly misidentified lines.

Animals and other research organisms

Policy information about studies involving animals; ARRIVE guidelines recommended for reporting animal research, and Sex and Gender in Research

Laboratory animals

C57BL/6J mice (ERG: age range 3-4 weeks, 5 mice, housed under standard conditions - 12-hour light/dark cycle, ambient temperature between 20-24 degrees centigrade, relative humidity between 45 to 65%; GPR156: three mice kept in 12-hour light/dark cycles, ambient temperature between 24 and 28 degrees centigrade with humidity between 45 to 65%).

Wild animals

The study did not involve wild animals.

Reporting on sex

Sex was not relevant to the study.

Field-collected samples

The study did not involve samples collected from the field.

Ethics oversight

University of Maryland, Baltimore Institutional Animal Care and use Committee (IACUC 420002) approved the study of GPR156. The study of ERG in mice were conducted with ethical approval from Imperial College London under UK Home Office Project Licence number PEDBB1586 in compliance with the UK Animals (Scientific Procedures) Act of 1986.

Note that full information on the approval of the study protocol must also be provided in the manuscript.

Clinical data

Policy information about clinical studies

All manuscripts should comply with the ICMJE guidelines for publication of clinical research and a completed CONSORT checklist must be included with all submissions.

Clinical trial registration	N/A
Study protocol	Refer to Genomics England Limited's website for information on data collection.
Data collection	Refer to Genomics England Limited's website for information on data collection.
Outcomes	N/A
