# Supplementary figures and images for: Genetic association analysis of 77,539 genomes reveals rare disease etiologies
Source: Nat Med. 2023 Mar 16;29(3):679–88. doi: 10.1038/s41591-023-02211-z (PMC10033407; doi:10.1038/s41591-023-02211-z)

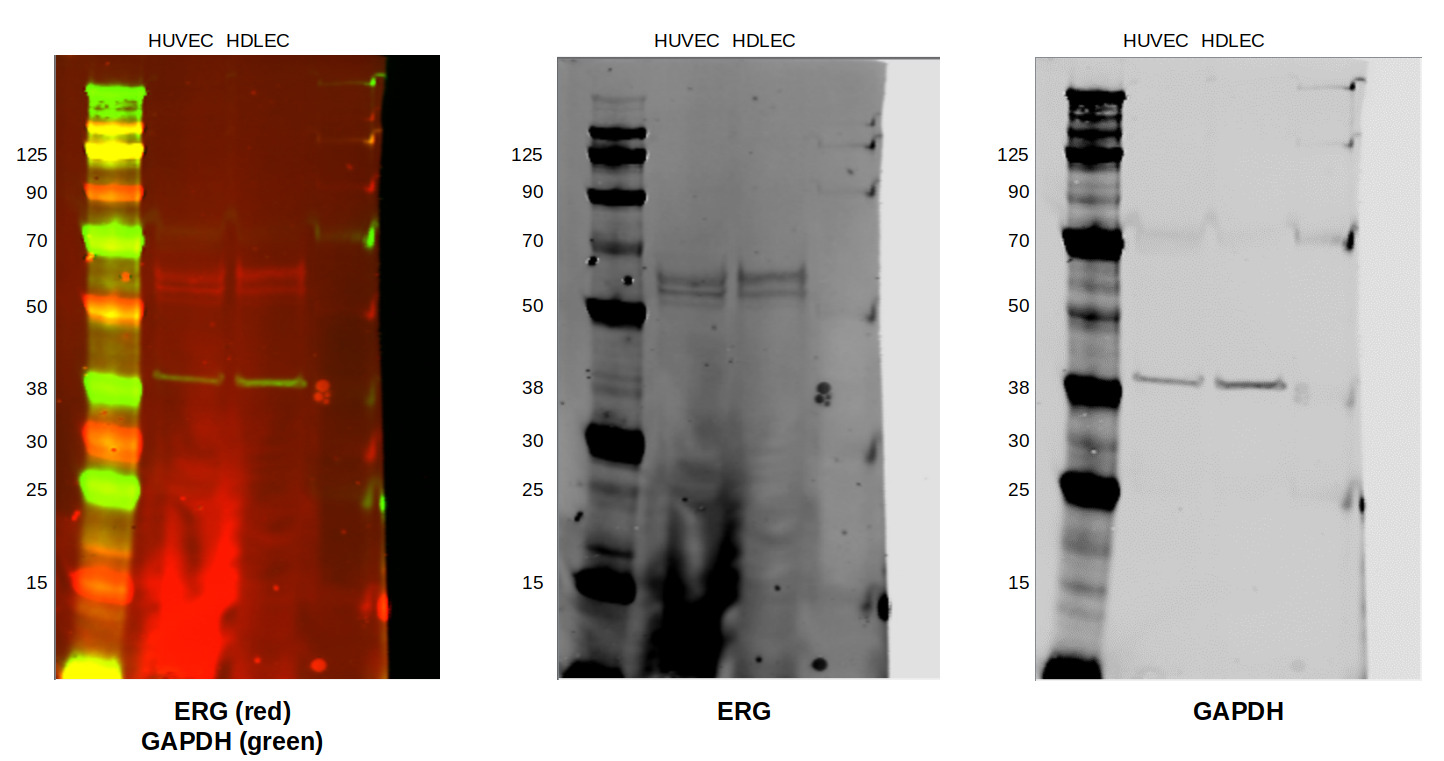

Supplement: Source Data Fig. 2 — Uncropped western blot images corresponding to Fig. 2e. [file 41591_2023_2211_MOESM3_ESM.jpg]

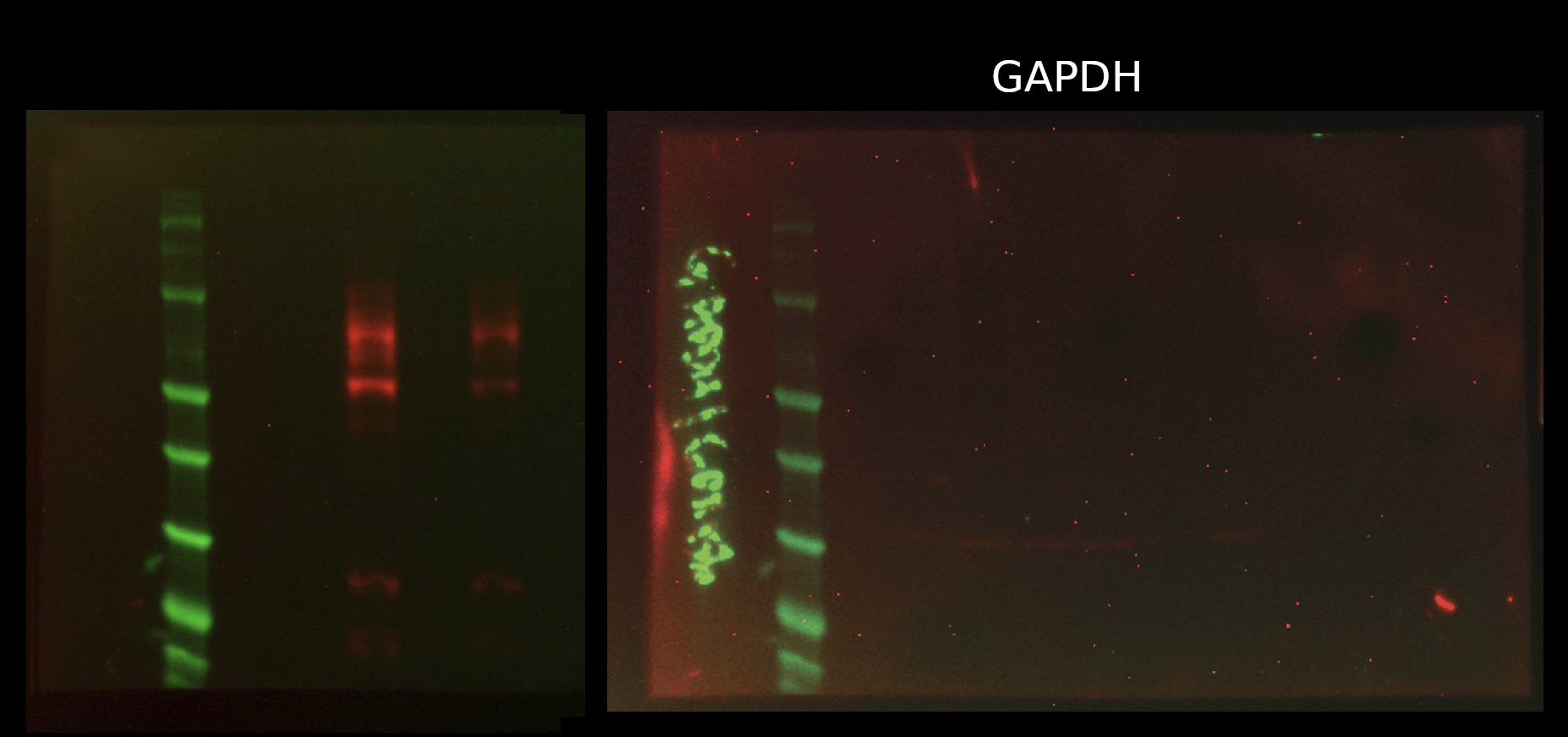

Supplement: Source Data Fig. 4 — Uncropped western blot images corresponding to Fig. 4e. [file 41591_2023_2211_MOESM4_ESM.jpg]
